# DrugScore^PPI^ Knowledge-Based Potentials Used as Scoring and Objective Function in Protein-Protein Docking

**DOI:** 10.1371/journal.pone.0089466

**Published:** 2014-02-21

**Authors:** Dennis M. Krüger, José Ignacio Garzón, Pablo Chacón, Holger Gohlke

**Affiliations:** 1 Institute for Pharmaceutical and Medicinal Chemistry, Heinrich-Heine-University, Düsseldorf, Germany; 2 Rocasolano Physical Chemistry Institute, Consejo Superior de Investigaciones Científicas, Madrid, Spain; German Research School for Simulation Science, Germany

## Abstract

The distance-dependent knowledge-based DrugScore^PPI^ potentials, previously developed for *in silico* alanine scanning and hot spot prediction on given structures of protein-protein complexes, are evaluated as a scoring and objective function for the structure prediction of protein-protein complexes. When applied for ranking “unbound perturbation” (“unbound docking”) decoys generated by Baker and coworkers a 4-fold (1.5-fold) enrichment of acceptable docking solutions in the top ranks compared to a random selection is found. When applied as an objective function in FRODOCK for bound protein-protein docking on 97 complexes of the ZDOCK benchmark 3.0, DrugScore^PPI^/FRODOCK finds up to 10% (15%) more high accuracy solutions in the top 1 (top 10) predictions than the original FRODOCK implementation. When used as an objective function for global unbound protein-protein docking, fair docking success rates are obtained, which improve by ∼2-fold to 18% (58%) for an at least acceptable solution in the top 10 (top 100) predictions when performing knowledge-driven unbound docking. This suggests that DrugScore^PPI^ balances well several different types of interactions important for protein-protein recognition. The results are discussed in view of the influence of crystal packing and the type of protein-protein complex docked. Finally, a simple criterion is provided with which to estimate *a prior*i if unbound docking with DrugScore^PPI^/FRODOCK will be successful.

## Introduction

Protein-protein interactions have important implications in most complex cellular signalling processes [1]. As a consequence, interfaces of protein-protein interactions are becoming increasingly important as drug targets [2], [3]. Several studies pointed to the existence of hotspot residues that account for most of the binding free energy in these interfaces [4], [5], [6], [7], [8]. These hotspots help guiding the development of modulators of protein-protein interactions [9]. For computational hotspot detection, most of the methods require knowledge of the protein-protein complex [2], [3], [10]. Likewise, structural knowledge of protein-protein complexes is valuable for understanding the complex connection between individual molecules and cell behavior [11]. Compared to the case of single protein structures, the number of experimentally determined structures of protein-protein complexes is still very limited. To overcome this limitation, various protein-protein docking approaches have been developed for predicting the structure of protein-protein complexes [12], [13], [14], [15], [16], [17].

Sampling of possible protein-protein configurations and scoring each configuration are the two main aspects for successful protein-protein docking. Whereas the configurational space of two (rigid) proteins can be successfully sampled in reasonable time, the reliable identification of near-native protein-protein complex structures from a set of generated configurations is still a major challenge [18]. At present, four types of functions to evaluate the quality of a predicted protein-protein configuration can be distinguished: I) based on physical force fields [19], [20]; II) based on shape complementary and additional descriptors related to desolvation or electrostatic interactions [21], [22]; III) empirical functions that are obtained by fitting to experimental data [23], [24], [25]; IV) knowledge-based potentials that are derived from databases of experimentally determined structures [26], [27], [28], [29]. Scoring functions can be further classified into residue-level potentials [30], [31] and atomic potentials [32], [33]. Residue-level (coarse-grained) potentials are computationally advantageous especially when applied to predict protein-protein complexes where the binding partners can undergo large conformational changes [34], [35], [36]. In contrast, atomic potentials are of higher resolution and are supposed to be most accurate and specific [37]. Atomic potentials are often knowledge-based; such potentials have been widely applied to score protein-ligand, protein-RNA, and protein-DNA interactions [38], [39], [40]. The reduced steepness of knowledge-based potentials compared to force field-based or empirical scoring functions has been recognized as an advantage in docking [41]. Regarding protein-protein interactions, only a few knowledge-based potentials have been described so far, including the contact potential IFACE [32] and two approaches using information from docking decoys as the knowledge base, the decoys-as-a-reference-state approach DARS [33] and the two-step potential TS [42]. The IFACE potential uses surface fraction information to define the reference state and is implemented as objective function in the Fast Fourier Transform (FFT)-based protein-protein docking program ZDOCK [32]. DARS is a statistical potential using information from interfaces of incorrect protein complex formations as an average reference state and is implemented as objective function in the FFT-based protein-protein docking program PIPER [43]. Both IFACE and DARS are used in combination with potentials for shape complementarity and electrostatics. The TS potentials were trained on protein-protein docking decoys by linear programming techniques and have been used as scoring functions [42], [44].

We recently developed the knowledge-based scoring function DrugScore^PPI^ for *in silico* alanine scanning and hot spot prediction on given structures of protein-protein complexes (accessible at http://cpclab.uni-duesseldorf.de/dsppi/) [10]. For this, distance-dependent pair-potentials were derived from 851 crystallographically determined protein-protein complexes. DrugScore^PPI^ is based on the DrugScore approach that has proven successful already as a scoring and objective function for protein-ligand [38], [45], [46], [47] and RNA-ligand [39], [48] complexes. In part, this has been attributed to the implicit, well-balanced consideration of several different types of interactions important for molecular recognition, such as polar (including hydrogen bonding), charged, and nonpolar interactions. Obtaining such a delicate balance is also considered crucial for successfully predicting protein-protein complexes [43], [49], [50].

This provided the incentive for us to evaluate the DrugScore^PPI^ potentials in structure prediction of protein-protein complexes. In this study, DrugScore^PPI^ was initially used as a scoring function to evaluate decoys of two non-redundant datasets of 54 protein-protein complexes by Gray *et al.* [15]. Subsequently, DrugScore^PPI^ was used as an objective function in connection with the fast spherical harmonics-based protein-protein docking algorithm FRODOCK [14]. To the best of our knowledge, this is the first time that atomic, distance-dependent, and knowledge-based potentials are used as the sole objective function (i.e., without any additional potential terms) in connection with an FFT-based docking approach, that way combining advantages of both methods in terms of sampling efficiency and scoring accuracy. The performance of DrugScore^PPI^ as an objective function was evaluated in bound and unbound docking on the ZDOCK benchmark 3.0 [51]. We discuss these results in view of the influence of crystal packing and the type of protein-protein complex docked. We too provide a simple criterion with which to estimate *a prior*i if unbound docking with DrugScore^PPI^/FRODOCK will be successful.

## Materials and Methods

### Distance-dependent Pair-potentials and Docking Score

The derivation of the distance-dependent pair-potentials of DrugScore^PPI^ was described in detail recently [10]. In short, the same formalism was applied as already used for the DrugScore and DrugScore^RNA^ scoring functions for protein-ligand and RNA-ligand complexes [38], [39]. The DrugScore^PPI^ potentials were derived from 851 crystallographically determined protein-protein complexes using an in-house mySQL database that contains structural information of all PDB entries (Grimme, D.; Radestock, S.; Schmidt, E.; Derksen, S.; Gohlke, H. unpublished results). The dataset consists of 655 homodimers and 196 heterodimers. In all of the cases, the complexes had been resolved to at least 2.5 Å. PDB codes of all complexes used for deriving the potentials are listed in ref. [29]. Potentials were derived for all DrugScore standard atom types that occur in the 20 canonical amino acids [38].

Summing over the resulting specific interactions Δ*W*(*T_p_, T_b_, d_p,b_*) between atom *p* with type Τ*_p_* of a protein P and atom *b* with type Τ*_b_* of the binding partner B, separated by a distance *d_p,b_*, results in the docking score Δ*W* for evaluating a protein-protein complex configuration (eq. 1). The upper distance for deriving the pair-potentials was set to 5 Å.
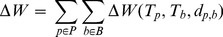
(1)


Contrary to previous work on alanine scanning [10], a scaling of the pair-potentials did not prove advantageous here. Thus, all results below were obtained with non-scaled Δ*W*(*T_p_, T_b_, d_p,b_*).

### Validating DrugScore^PPI^ Potentials as a Scoring Function

Initially, the predictive power of the DrugScore^PPI^ potentials was assessed in terms of their ability to select native-like protein-protein complex configurations from datasets of pre-generated protein-protein docking decoys. Thus, the DrugScore^PPI^ potentials were evaluated as a scoring function. For this we used two non-redundant datasets constructed by the Baker group using the protein-protein docking program RosettaDock [15]. Hence, results obtained with DrugScore^PPI^ for these complexes can be directly compared to the validation study by Baker and coworkers [15]. The first dataset consists of 54 complexes for which 1000 “unbound perturbation solutions” have been generated, respectively; the second one consists of 54 complexes for which 200 “unbound docking solutions” have been generated, respectively. Here, “unbound perturbation solutions” refers to complex structures generated from the binding partners in an unbound conformation by sampling around the native ligand position; “unbound docking solutions” refers to a complete sampling of the global search space. All structures of the decoy sets have optimized side chain conformations, and their energies were minimized to reduce steric clashes, i.e. the decoy structures are stereochemically correct. For a more detailed description of the decoy generation see ref. [15]. Each decoy was rescored according to eq. 1, and the decoys of one protein-protein complex were ranked according to the scores. The larger protein (referred to as the receptor) was considered protein P according to eq. 1, and the smaller protein (referred to as the ligand) as binding partner B. Rankings for the “unbound perturbation solutions” were evaluated by calculating the percentage of complexes that have at least one solution, or at least three solutions, with an all-atom rmsd <10 Å in the top 5 scoring ranks, respectively. For “unbound docking solutions” complexes were first clustered by a threshold of 2.5 Å using the kclust algorithm from the AMBER suite of programs [52]. These clusters were then sorted according to the cluster size with the largest cluster getting the best rank. Finally, solutions were obtained considering the best scored solution of each of the clusters, respectively. The rankings were evaluated by calculating the percentage of complexes that have at least one solution with an all-atom rmsd <5 Å or <10 Å in the top 10 scoring ranks. These clustering and ranking criteria are according to the work of Baker and coworkers [15].

### Integrating DrugScore^PPI^ Potentials as an Objective Function into the Docking Program FRODOCK

FRODOCK is a fast spherical harmonics-based protein-protein docking tool developed by Garzón *et al.* [14]. The original implementation uses potential grids encoding van der Waals, electrostatic, and desolvation energies to score the predicted complexes. Accordingly, FRODOCK was extended to use pre-calculated DrugScore^PPI^ potential grids for approximating the binding energy upon complex formation Δ*W* (eq. 1). For distances *d_p,b_* smaller than the location of the first maximum of a pair-potential with respect to the origin plus 0.1 Å, a Gaussian repulsion term with a height of 280.000 at *d_p,b_* = 0 Å was added to the DrugScore^PPI^ pair potentials as described in ref. [47]. This ensures that repulsive forces act between a protein and its binding partner at short distances for which no information is available in the database used for deriving the DrugScore^PPI^ potentials. Each rectangular potential grid was located at the center of mass of a receptor. The size of the grid in the {x, y, z} direction was determined from the maximum difference of the {x, y, z} coordinates of two atoms plus the upper distance limit of the pair-potentials of 5 Å. The rotational and translational sampling resolutions were set to 5.6° (∼60.000 rotations) [14] and 1 Å, respectively.

### Validating the DrugScore^PPI^/FRODOCK Approach

For validating the DrugScore^PPI^/FRODOCK approach, we used protein-protein complexes from the ZDOCK benchmark 3.0 prepared by Hwang *et al.* [51]. The benchmark consists of 124 protein-protein complexes for which bound-bound and unbound-unbound binding partners are available. For 10 of the complexes only one of the two binding partners is in an unbound conformation. Complex predictions were evaluated based on interface (i_rmsd) and ligand (l_rmsd) backbone root mean square deviations as well as the fractions of native (f_nat_) and non-native (f_not_) contacts of interface residues following CAPRI criteria [53]. Based on these parameters, the quality of predictions is classified as high accuracy (f_nat_ ≥0.5 and (l_rmsd ≤1.0 Å or i_rmsd ≤1.0 Å)), medium accuracy ((0.3≤ f_nat_ ≤0.5) and (l_rmsd ≤5.0 Å or i_rmsd ≤2.0 Å) or (f_nat_ >0.5 and l_rmsd >1.0 Å and i_rmsd >1.0 Å)), acceptable accuracy ((0.1≤ f_nat_ ≤0.3) and (l_rmsd ≤10.0 Å or i_rmsd ≤4.0 Å) or (f_nat_ >0.3 and l_rmsd >5.0 Å and i_rmsd >2.0 Å)), and incorrect (f_nat_ <0.1 or (l_rmsd >10.0 Å and i_rmsd >4.0 Å)) according to ref. [29]. A receptor or ligand residue is considered in the interface if any of its atoms is within 10 Å of any atom of the ligand or the receptor, respectively. Interface residue contacts are defined in the same way but using a distance of 5 Å. The complexes from the ZDOCK benchmark 3.0 can be grouped into three categories with respect to conformational changes occurring between unbound and bound state of the binding partners: easy (C_α_ -i_rmsd <1.5 Å and f_not_ <0.4), medium (1.5 Å<C_α_-i_rmsd ≤2.2 Å, or C_α_ -i_rmsd <1.5 Å and f_not_ >0.4), and difficult (C_α_ -i_rmsd >2.2 Å) cases [51]. All proteins were checked manually for missing or incomplete residues in the native protein-protein interface. Single missing sidechains were reconstructed using PyMol [54] choosing the most favourable rotamer that does not show steric clashes. Two or three sequentially adjacent residues either missing or being incomplete were reconstructed using Maestro [55]. 27 benchmark entries missing in at least one of the two binding partners more than three sequentially adjacent residues in the native protein-protein interfaces were skipped (Table S1 in File S1). Thus, our benchmark finally consists of 97 protein-protein complexes. Examples for skipped entries are depicted in Figure S1 in File S1; the missing residues are mostly located in the binding partner(s) that are in the unbound conformation. It can be expected that in these cases the docking result will be critically influenced by the missing residues.

The final benchmark set shares five homologous complexes with the knowledge base of 851 structures from which the DrugScore^PPI^ potentials were derived. To test whether this leads to a training effect on the potentials, we re-derived the DrugScore^PPI^ potentials from the knowledge base excluding the homologous complexes. When applied in the evaluation studies, the re-derived potentials did not lead to significantly different results (data not shown). This observation is consistent with results from a leave-homologous-complexes-out cross-validation study when applying the DrugScore^PPI^ potentials for *in silico* alanine-scanning [10]. This points to the robustness of the derived DrugScore^PPI^ potentials.

### Computational Efficiency of DrugScore^PPI^/FRODOCK

The docking with DrugScore^PPI^/FRODOCK (without considering the precalculation of the potential grids) was evaluated with respect to computational efficiency on three protein-protein complexes with different sizes (Table S2 in File S1). Using 16 cores on dual CPU compute servers equipped with 2 GHz Intel Xeon Quadcore CPUs, 24 GB of RAM, and Infiniband interconnect, a docking run takes ∼17 minutes for the small complex, ∼22 minutes for the medium complex, and ∼4.5 hours for the large complex. Using either 8 cores or 32 cores reduced the computational efficiency because of too little memory in the former case or communication overhead in the latter case. A drastic decrease (∼13-fold) in the efficiency was observed on going from the medium system to the large system. This can be explained by the ∼2-fold larger ligand and the ∼5-fold increased search spaced related to the ∼1.7-fold difference in the maximum diameters of the receptors. Compared to the original FRODOCK implementation, the efficiency of the DrugScore^PPI^/FRODOCK combination is ∼2.2-fold decreased (Table S2 in File S1). This difference can be explained by the number of eleven grid maps used in the case of DrugScore^PPI^/FRODOCK compared to only four grid maps in the case of original FRODOCK, resulting in a larger computational burden for energy evaluations in the former case.

## Results and Discussion

### Characteristics of the Distance-dependent Protein-protein Atom Pair-potentials

The distance-dependent pair-potentials of DrugScore^PPI^ were derived using the same formalism as already applied for the DrugScore [38] and DrugScore^RNA^ [39] pair-potentials for scoring protein-ligand and RNA-ligand complexes, respectively. Previously, DrugScore^PPI^ was successfully applied for computational alanine scanning [10]. Here, we will characterize the DrugScore^PPI^ pair-potentials with respect to their suitability for scoring protein-protein complex configurations.

**Figure 1:**
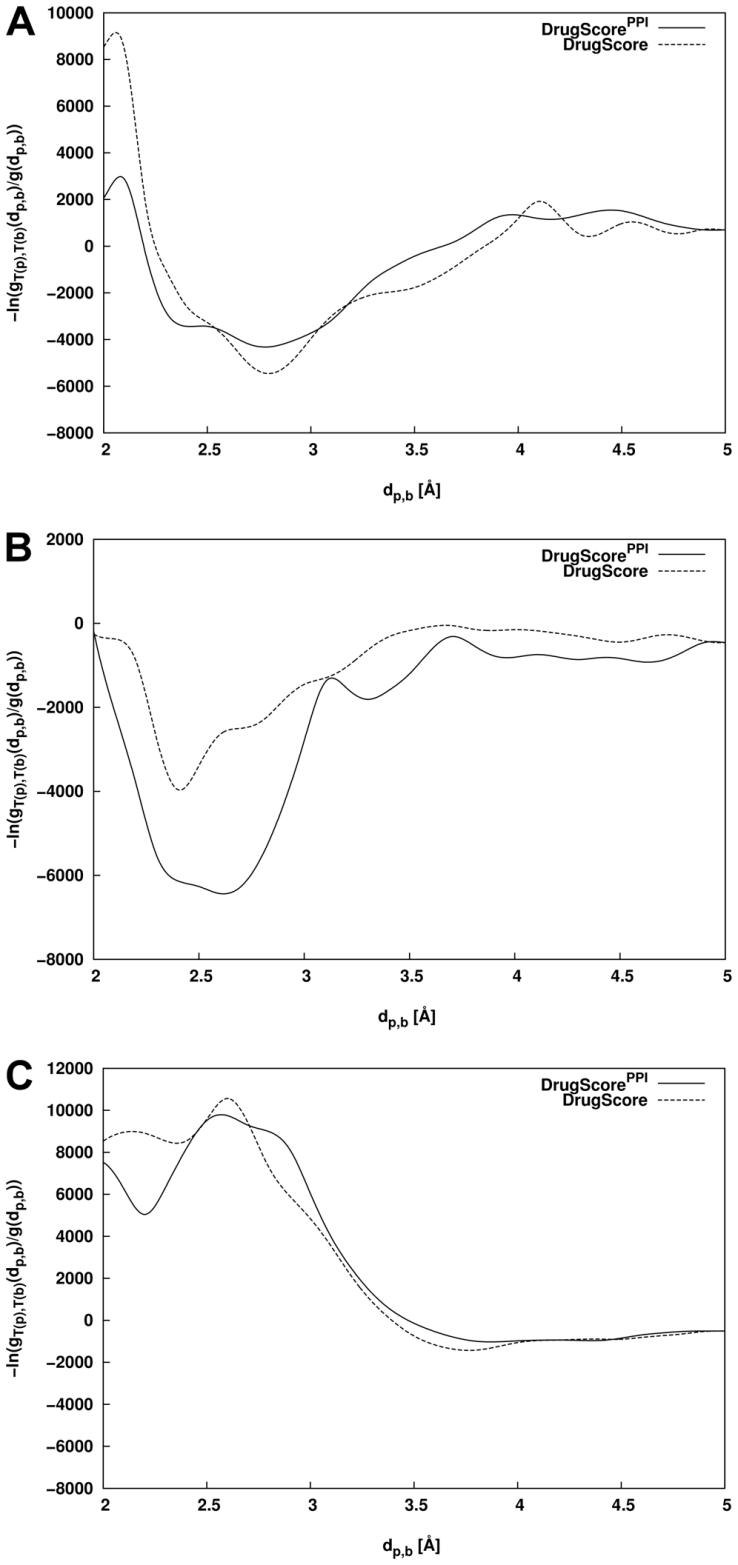
Distance-dependent pair-potentials of DrugScore^PPI^ (straight line) and DrugScore [38] (dashed). (A) Charged interactions between atoms of types N.pl3 and O.co2; (B) polar interactions between two atoms of type O.3; (C) aromatic interactions between two atoms of type C.ar. For reasons of comparison, the potentials were aligned to a value of zero at a distance of 5 Å.

Previous experience indicates that at least 500 interactions (i.e., about 10 interactions per distance bin) are required per atom−atom pair to obtain statistically significant potentials [38], [56]. This requirement is fulfilled for the examples of pair-potentials depicted in Figure 1 (pair-potential N.pl3-O.co2∶2923 interactions; O.3-O.3∶831; C.ar-C.ar: 12833). For a list of all atomtypes considered in DrugScore^PPI^ see Table S3 in File S1. Over all 121 pair-potentials derived, the least number of interactions was found for the potential S.3-N.3 (10), whereas the most interactions were found for C.3-C.ar (34333). Less than 500 interactions were found between N.3 and positively charged atomtypes as well as for S.3 and positively or negatively charged atomtypes. However, such interactions are rather unlikely to occur when evaluating protein-protein complex configurations, too, and thus should not grossly affect the scoring results. These results indicate that the knowledge base of 851 protein-protein complexes for derivation of DrugScore^PPI^ is large enough to yield statistically significant potentials despite the smaller number of complexes used for deriving DrugScore^PPI^ than for deriving DrugScore [38]. Two reasons account for this: first, the number of pair interactions per complex is much larger in the case of protein-protein complexes than in the case of protein-ligand complexes due to the larger size of the binding partners; second, in the case of DrugScore^PPI^, pair-potentials do not need to be derived for rarely occurring ligand atoms such as halogens.

The pair-potentials shown in Figure 1 are representative for interactions between charged atoms (N.pl3 vs. O.co2; Figure 1A), polar interactions (O.3 vs. O.3; Figure 1B), and aromatic interactions (C.ar vs. C.ar; Figure 1C) The respective potentials from DrugScore and DrugScore^PPI^ show qualitatively similar shapes but quantitative differences with respect to the minima in the case of N.pl3-O.co2 and O.3-O.3. As such, for N.pl3-O.co2 interactions both a global minimum (at (d, *ΔW*) values of (2.8 Å, −4314)) and a local minimum (2.3 Å, −3435) were found in the case of DrugScore^PPI^ but only a global minimum in the case of DrugScore (2.8 Å, −5458). Both the global minima reflect the typical distance for hydrogen bonding including salt bridges [56]. The short distance of the local minimum might be explained by the formation of tight hydrogen bonding interaction networks in protein-protein interfaces that is induced by a close packing of the interface residues [28]. Visual inspection in the database used for DrugScore^PPI^ derivation confirmed that indeed for many salt bridges between Asp or Glu and Arg one of the N.pl3-O.co2 interactions is shorter than the other one. Hence, the minima of the DrugScore^PPI^ potential reflect the ability of proteins to diversify salt bridge interactions as a result of residue packing. The O.3-O.3 interactions at the minimum around 2.5 Å are much more favorable in DrugScore^PPI^ (Δ*W*≈−6434) than in DrugScore (Δ*W*≈−3961). This is an interesting result because in the case of protein-protein interactions there are only three residues, Thr, Ser, and Tyr, that are able to form O.3-O.3 interactions. Regarding Ser and Thr, there is a need for small residues for tight interface packing; thus, the multiple polar interactions of Ser and Thr can appreciably contribute to protein-protein binding. Tyr is one of the most common hotspot residues in protein interfaces as it can form aromatic interactions in addition to hydrogen bonds [7]. Finally, C.ar-C.ar interactions (*ΔW*_DrugScore_PPI_ = −1023; *ΔW*_DrugScore_ = −1419) are almost similar in both potentials.

In summary, the DrugScore^PPI^ potentials encode characteristic determinants of the molecular recognition of proteins, which differ from those observed in DrugScore pair potentials of protein-ligand interactions. This indicates that these knowledge-based potentials do not have a universal character; rather, the respective potential can be expected to show a high predictive power only when applied to cases that lie within the scope of the knowledge base used for its derivation. The generalizability of the potentials is expected to increase with the size of the knowledge base they are derived from even if the current knowledge base already yielded statistically significant potentials (see above). Considering that the derivation of DrugScore^PPI^ potentials occurs in an automated manner, this suggests to re-derive a new DrugScore^PPI^ version once the number of protein-protein complexes has increased markedly in the PDB. In addition to extending the knowledge base of native complexes, the knowledge base can also be extended by considering non-native complexes, i.e., docking decoys for using DrugScore^PPI^ potentials as a scoring and objective function in structure prediction of protein-protein complexes. Applying linear programming, the pair-potentials can then be scaled with the objective to maximize the gap between scores of native *versus* non-native complexes [57].

### Scoring of Decoy Sets of Protein-protein Complexes

In a first step, DrugScore^PPI^ was used as a scoring function for ranking decoys of a non-redundant dataset of 54 targets for which “unbound perturbation” and “unbound docking” solutions have been generated by Baker and coworkers (Table S4 & S5 in File S1) [15]. For the “unbound perturbation” dataset, on average 28% of the generated decoys have an all-atom rmsd <10 Å from the native structure (Table S4 in File S1); for the “unbound docking” dataset, this holds for on average 11% of the decoys. More severe, the “unbound docking” decoy set contains 17 (7) targets for which no (only one) decoy with an all-atom rmsd <10 Å was generated (Table S5 in File S1). For these targets it will be impossible (very difficult) to identify an acceptable decoy (all-atom rmsd <10 Å) by rescoring. Thus, we separately evaluated the rescoring results for those “unbound docking” decoy sets where at least two acceptable decoys are available. Considering this subset, on average 19% of the generated decoys have an all-atom rmsd <10 Å from the native structure. This number drops to 0.4% for the subset where less than two acceptable decoys are available with an all-atom rmsd <10 Å from the native structure (Table S5 in File S1).

When scoring the “unbound perturbation” dataset, DrugScore^PPI^ was able to rank at least one (three) solution(s) with rmsd <10 Å in the top 5 in 81.5% (57.4%) of the cases (Table 1). Detailed results are given in Table S6 in File S1. Considering that on average 28% of the “unbound perturbation” decoys have an all-atom rmsd <10 Å, the probability to rank at least one (three) solution(s) in the top 5 by random selection is 81% (14%). The latter result shows that DrugScore^PPI^ yields a 4-fold enrichment of acceptable docking solutions in the top ranks compared to a random selection. When applied to the dataset of “unbound docking” solutions, DrugScore^PPI^ was able to rank a solution in the top 10 with rmsd <10 Å (5 Å) in 100% (73.3%) of the cases (Table 1). Detailed results are given in Table S7 in File S1. The probability to rank a solution with rmsd <10 Å (5 Å) in the top 10 by random selection is 88% (48%) for the decoy sets containing at least two acceptable decoys with an all-atom rmsd <10 Å from the native structure. For the decoy sets containing less than two acceptable decoys with an all-atom rmsd <10 Å from the native structure this probability is <1% (<1%). For these cases, DrugScore^PPI^ yields 25.0% (20.8%). Thus, when compared to the probabilities for random selection, DrugScore^PPI^ shows superior performance in ranking acceptable solutions on the top. Comparing these results to the ones of Baker and coworkers (Table 1) shows that DrugScore^PPI^ performs slightly inferior in the case of the “unbound perturbation” dataset but superior in the case of the “unbound docking” dataset. This result is remarkable given that the scoring function has been derived based on a formalism originally established for protein-ligand interactions and that no tweaking of parameters with respect to scoring protein-protein complexes has been done. Given the ease with which DrugScore^PPI^ can be derived, it is worth testing if its predictive power can be increased further by re-deriving the function on extended datasets in the future. The result also suggests that DrugScore^PPI^ should be suitable as an objective function for protein-protein docking.

**Table 1:** Results of scoring decoys from the dataset of Baker and coworkers [15].

Criterion	Unbound perturbation[a]		Unbound docking[b]	
	This work	Baker and coworkers	This work	Baker and coworkers
R5Å	−[c]	−[c]	73.3 (20.8)	66.7 (20.8)
R10Å	−[c]	−[c]	100.0 (25.0)[d]	93.3 (25.0) [d]
N10Å	57.4	63.0	−[c]	−[c]
Best rmsd	81.5	83.3	100.0 (25.0) [d]	93.3 (25.0) [d]

[a] “Unbound perturbation” dataset. 54 targets were scored with 1000 decoys each. Scoring criteria were applied according to Baker and coworkers: “N10Å” is the percentage of complexes that have at least three top five decoys with rmsd <10 Å; “Best rmsd” is the percentage of complexes that have at least one top five decoy with rmsd <10 Å. Results from this work and the study by Baker and coworkers are shown.

[b] “Unbound docking” dataset. 54 targets were scored with 200 decoys each. To identify the top 10 solutions, the best scored decoys from the top 10 clusters were considered (see Materials & Methods). Scoring criteria were applied according to Baker and coworkers: “R5Å” (“R10Å”) is the percentage of complexes that have at least one solution <5 Å (<10 Å) in the top 10 decoys. “Best rmsd” is the percentage of complexes that have at least one solution with rmsd <10 Å in the top 10 decoys. Numbers not in parentheses refer to the 30 targets for which at least two “good” decoys are available; numbers in brackets refer to the other 24 targets. Results from this work and the study by Baker and coworkers are shown.

[c] Not determined.

[d] For reasons of comparison with the paper of Baker and coworkers both values are given although they are redundant.

### Analysis of Binding Energy Landscapes

In order to further analyze the properties of DrugScore^PPI^, we investigated the binding energy landscapes of the 54 complexes of the “unbound perturbation” dataset with 1000 decoys each. A successful representation of protein-protein interactions should not only allow a reliable recognition of near-native docking solutions but should also produce a binding (free) energy landscape that is smooth as to not impair the efficiency of configurational sampling during docking [39]. In that respect, funnel-shaped binding (free) energy surfaces of protein-protein complex formation are expected, similar to what is known from the field of protein-ligand docking [58], [59], [60]. In previous studies, the Spearman correlation coefficient *R*S was used as a quantitative measure to determine the correlation between the rmsd values and the scores of docking solutions [39], [61]. Although not sufficient to comprehensively define the funnel-shapeness of the energy landscape due to its high-dimensional character, such a correlation is assumed to be at least necessary for a funnel to exist. Here, we adopt the same measure.

**Figure 2:**
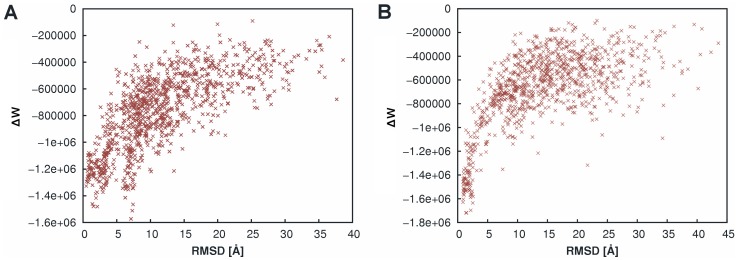
Computed scoring values of decoys from the “unbound perturbation” dataset using DrugScore^PPI^. (A) Serine protease/prosegment complex (PDB-ID 1PPE); (B) trypsin/trypsin inhibitor complex (PDB ID 1SPB). The scoring values are given as a function of the rmsd from the native structure; small rmsd values denote near native-like protein-protein configurations.

In Figure 2 the DrugScore^PPI^ scores for 1000 “unbound perturbation” decoys of a serine protease/prosegment complex (PDB-ID 1PPE) and a trypsin/trypsin inhibitor complex (PDB ID 1SPB) are shown as a function of the rmsd with respect to the native structure. In both cases, a well-defined funnel-shape is obvious, although in the former case a decoy with rmsd = 7.11 Å is slightly favored over more near-native solutions. The *R*S is 0.75 (0.61) for the protease/prosegment complex (trypsin/trypsin inhibitor complex). When considering all 54 complexes, for 59% (44%) an *R*S of at least 0.3 (0.4) was found (Figure S2 in File S1). These results underscore the reduced steepness of the knowledge-based DrugScore^PPI^ potentials, which has been recognized as an advantage in small-molecule/receptor docking studies [39], [41].

### Using DrugScore^PPI^ as Objective Function for Bound Protein-protein Docking with FRODOCK

DrugScore^PPI^ was initially used as objective function in FRODOCK to predict 3D structures of protein-protein complexes from conformations of the binding partners in the bound state. As conformational changes of the binding partners are neglected in this step, this approach allows determining under best conditions to what extent the objective function/docking tool combination is able to generate and discriminate (near-)native binding modes.

For our purpose, FRODOCK was adapted such that precalculated DrugScore^PPI^ potential grids can be used as input. Eleven potential grids (Table S3 in File S1) were calculated using the larger binding partner as “receptor”; configurations of the smaller binding partner (“ligand”) were sampled then during docking. For comparison, docking with the original FRODOCK implementation was performed, too, applying standard parameters suggested by Garzón *et al.* [14] except for the step size of the translational search, which was reduced from 2 Å to 1 Å to improve the sampling density. For the docking with DrugScore^PPI^ potential grids, we used the same settings. FRODOCKCLUST was used to cluster the predicted complex configurations with a threshold of 5 Å rmsd. For each docking run, of all the best-scored solutions from each of the clusters, the 2000 top-ranked ones were finally evaluated with respect to their structural quality. This quality was assessed following CAPRI criteria (see Materials and Methods section) [53]. Note that for 11 protein-protein complexes multiple ligand binding modes need to be considered for evaluation (Table S8 in File S1). Multiple binding modes were identified by visual inspection of the original PDB files.

When applied to a subset of 97 bound test cases of the ZDOCK benchmark 3.0 (see Materials and Methods section), convincing results were obtained with the DrugScore^PPI^/FRODOCK combination (Table 2 and Table S9 in File S1). A high or medium accuracy solution in the top 1, top 10, and top 100 rank(s) was obtained in 53.1%, 69.8%, and 80.2% of the cases, respectively. Compared to the original FRODOCK implementation (Table 2 and Table S10 in File S1), DrugScore^PPI^/FRODOCK led to docking success rates that are higher by >10% for the top 1 and top 10 ranks. In particular, DrugScore^PPI^/FRODOCK is able to find up to 10% and 15% more high accuracy solutions in the top 1 and 10 predictions, respectively. Interestingly, hardly any acceptable solution was found; thus, docking solutions were either highly correct or incorrect.

**Table 2:** Success rates for bound docking using DrugScore^PPI^/FRODOCK, the original FRODOCK implementation, and rescoring original FRODOCK results with DrugScore^PPI^.^[a].^

Accuracy	DrugScore^PPI^/FRODOCK				FRODOCK				Rescoring FRODOCK		
	Top 1	Top 10	Top 100	Top 2000	Top 1	Top 10	Top 100	Top 2000	Top 1	Top 10	Top 100
High	15.6	20.8	22.9	24.0	5.2	5.2	10.4	11.5	0.0	0.0	1.0
Medium	37.5	49.0	57.3	68.8	34.4	53.1	70.8	81.3	1.0	2.1	16.7
Acceptable	0.0	0.0	0.0	1.0	0.0	0.0	1.0	1.0	1.0	6.3	32.3
Totals	53.1	69.8	80.2	93.8	39.6	58.3	82.3	93.8	2.0	8.4	50.0

[a] Docking calculations were performed for a subset of 97 structures of the ZDOCK benchmark 3.0 (see Materials and Methods section). The percentage of complexes is reported for which at least one solution with the given accuracy was found in the top 1, 10, 100, or 2000 solutions.

Antigen-antibody complexes have been found to show major differences in the interactions compared to other protein-protein complexes [62], [63], [64]. Our subset of test cases contains 23 antigen-antibody complexes. For ten of these complexes, no solution with at least acceptable accuracy was found in the top 10 for bound docking with DrugScore^PPI^/FRODOCK. Ramaraj *et al.* reported that Tyr shows the highest abundance of all amino acids in the paratope-containing surface (PCS) of an antibody and also the highest presence in the PCS compared to the surface of the whole antibody [65]. Accordingly, when sorting the top 100 predictions of a complex by their abundance of Tyr residues in the antibody interface, the number of failures decreased to seven (three) when considering the top 10 (20) ranks. These results suggest that it may be advantageous to derive knowledge-based pair potentials specifically for antigen-antibody complexes. For five protein-protein complexes, no near-native solution could be found at all by DrugScore^PPI^/FRODOCK: Three of them are antigen-antibody complexes (see above; PDB-IDs: 1E6J, 1I9R, 2HMI), and two are other complexes (PDB-IDs: 1GLA, 1I4D). We will discuss these failures in more detail in the section “Influence of crystal packing”.

Finally, when using DrugScore^PPI^ for rescoring the 2000 top-ranked decoys generated with the original FRODOCK implementation, a dramatic drop-off in the docking success rate was observed (Table 2 & Table S9 in File S1). A solution with at least acceptable accuracy in the top 1, top 10, and top 100 rank(s) was obtained in only 2.0%, 8.4%, and 50.0% of the cases, respectively. DrugScore^PPI^ and the FRODOCK scoring function apparently favor sufficiently different protein-protein complex configurations as near-native solutions such that rescoring only a subset of all FRODOCK-generated configurations with another scoring function fails. In turn, this stresses the importance of a thorough sampling of complex configurations as a prerequisite for accurate scoring, which is done implicitly when docking with either the DrugScore^PPI^/FRODOCK combination or the original FRODOCK implementation.

### Influence of Crystal Packing

Although X-ray crystallography is the most widely used method for structural investigations of complexes involving biomolecules [66], there has always been concerns whether the crystalline state influences structure and dynamics of such complexes [67]. This led us to investigate to what extent protein-protein docking results are affected by crystal packing contacts observed for the native complex structures. For this, we visualized the crystal environment of a protein-protein complex using Maestro [68] for all cases where docking with DrugScore^PPI^/FRODOCK was not successful in a docking experiment with bound-bound cases. The influence of crystal packing has been widely ignored so far when preparing benchmark sets of protein-protein complexes and in protein-protein docking studies.

**Figure 3:**
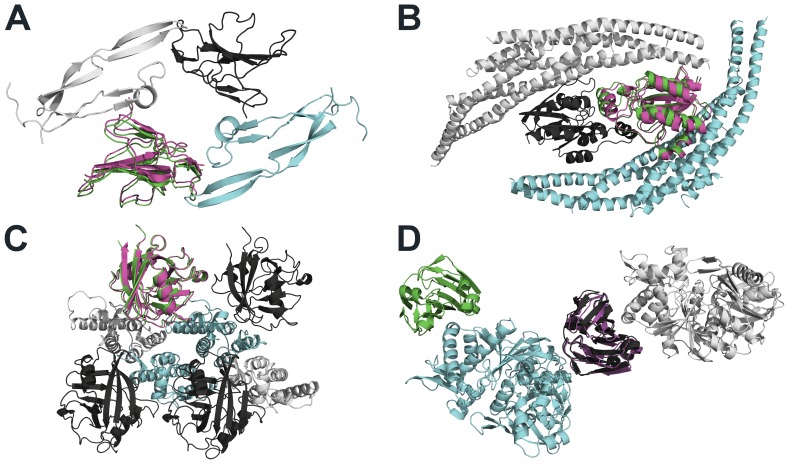
Biologically relevant protein-protein complexes and non-specific protein-protein interactions arising from crystal contacts. The receptor (ligand) in protein-protein complexes provided in the ZDOCK benchmark 3.0 is colored in cyan (green); receptor (ligand) molecules arising from crystal contacts are colored in white (black). Docking solutions are depicted in magenta. (A) Extracellular domain of the human TGF-beta type II receptor complexed with TGF-beta3 (PDB-ID 1KTZ). The docking solution was found on rank 6 when both receptor structures were considered for the docking. (B) RAC1-GDP complexed with ligand arfaptin (PDB-ID 1I4D). The docking solution was found on rank 3 when both receptor structures were considered for the docking. (C) Human cyclophillin A complexed with the amino-terminal domain of the HIV-1 capsid (PDB-ID 1AK4). The docking solution was found on rank 4 when a set of three receptor structures were considered for the docking. (D) *E. coli* IIIGlc complexed with glycerol kinase (PDB-ID 1GLA). The docking solution was found on rank 1 although only the native receptor was considered for the docking.

In the following, we will discuss four examples where crystal packing effects had a severe impact on our protein-protein docking results (Figure 3): I) For the complex of human TGF-beta type II receptor with TGF-beta3 (PDB-ID 1KTZ), no near-native solution was found in the top 10 when using the complex structure from the benchmark set as a reference. Regarding the crystal packing, the ligand has contacts to two receptor proteins, which are related by a two-fold rotation axis (Figure 3A). When both receptor structures were considered for protein-protein docking, a medium accuracy solution was found on rank 6. Notably, another crystal structure (PDB-ID 3KFD) already implicitly shows the effect of the crystal packing [69]. II) For the complex of RAC1-GDP with the ligand arfaptin (PDB-ID 1I4D), no near-native solution could be sampled at all. Regarding the crystal packing, two ligand structures are in contact with each other, and, in addition, each ligand has contacts to two receptor proteins (Figure 3B). When both receptor structures were considered for protein-protein docking, we found a medium accuracy solution on rank 3. Crystal lattice contacts for RAC have already been described by the authors that determined the complex structure [70]. III) For the complex of the amino-terminal domain of the HIV-1 capsid with human cyclophillin A (PDB-ID 1AK4), no near-native solution was found in the top 10 when using the complex structure from the benchmark set as a reference. Regarding the crystal packing, receptors and ligands share multiple interfaces: Each receptor structure is in contact with three other receptor structures, and each ligand binds to a set of three receptors (Figure 3C). When such a set of three receptors was considered for protein-protein docking, we were able to find a high accuracy solution on rank 4. It has already been described by the authors that several amino-terminal domains of the HIV-1 capsid associate into planar strips within the crystal consistent with what is depicted in Figure 3C [71]. IV) For the complex of *E. coli* IIIGlc with glycerol kinase (PDB-ID 1GLA), no near-native solution could be sampled among the top 2000. Interestingly, however, a solution was identified on the first rank that has medium accuracy with respect to a structural arrangement that does not result in the biologically relevant interaction but one originating from crystal contacts (Figure 3D). This finding reflects that non-specific protein-protein interactions make use of the same forces that govern specific recognition in protein-protein complexes [72]. Scoring schemes particularly trained on specific protein-protein interactions versus non-specific ones could be used as a postfilter for the DrugScore^PPI^/FRODOCK output to distinguish such cases [73].

In summary, these examples reveal that many “failures” in protein-protein docking can be understood if crystal packing effects are considered. Conversely, in our view, in the design of benchmarks for protein-protein docking, such potential influences should be taken into account, as has been done in the field of protein-ligand docking for the CCDC/Astex clean set [74]. Over and above considering the influence of additional, or alternative, interaction partners due to the crystal packing, one would also need to investigate whether side chain, loop, or even global conformations of the binding partners have been influenced by the crystalline state. Molecular dynamics simulations have been successfully used in this context to investigate the flexibility of protein structures prior to docking [75], [76], [77].

### Using DrugScore^PPI^ as Objective Function for Unbound Protein-protein Docking with FRODOCK

Docking proteins in the unbound conformation is considered difficult because pronounced conformational changes of the binding partners can occur upon complex formation that invalidate the lock-and-key principle underlying rigid protein-protein docking [78], [79], [80]. We tested the DrugScore^PPI^/FRODOCK approach again on the cleaned version of the ZDOCK benchmark 3.0, now using both protein binding partners in the unbound conformation (Table 3; Table S11 in File S1). The quality of the results was assessed as before for bound docking. Compared to bound docking (Table 2), a sharp drop in the success rates is observed. First, neither is a high accuracy complex structure found in the top 100 nor is one generated at all. Second, complex structures of medium and acceptable accuracy are found in the top 10 (top 100) in 8.3% (31.3%) of the cases. When only considering the easy cases (see Materials and Methods section for the definition), this success rate increases to 11.0% (39.7%) in the top 10 (top 100).

**Table 3:** Success rates for unbound docking using DrugScore^PPI^/FRODOCK and the original FRODOCK implementation.[a]

Accuracy	DrugScore^PPI^/FRODOCK[b]				DrugScore^PPI^/FRODOCK[c]				FRODOCK[b]			
	Top 1	Top 10	Top 100	Top 2000	Top 1	Top 10	Top 100	Top 2000	Top 1	Top 10	Top 100	Top 2000
Medium	5.2	6.3	14.6	33.3	6.3	10.4	25.0	49.0	7.3	13.5	29.2	59.4
Acceptable	0.0	2.1	16.7	34.4	0.0	7.3	33.3	33.3	3.1	9.4	17.7	21.9
Totals	5.2	8.3	31.3	67.7	6.3	17.7	58.3	83.3	10.4	22.9	46.9	81.3

[a] Docking calculations were performed for a subset of 96 structures of the ZDOCK benchmark 3.0 (see Materials and Methods section). The percentage of complexes is reported for which at least one solution with the given accuracy was found in the top 1, 10, and 100 solutions. The “Top 2000” column reports the percentage of complexes for which at least one solution with the given accuracy was found in the top 2000 solutions. In neither docking approach was a high accuracy solution found.

[b] A global search of ligand configurations around the receptor was performed as in the case of bound docking.

[c] The search space for the knowledge-driven docking was restricted to 10 Å around a central point. For details, see Figure S3 in File S1. The mean is reported for three independent docking runs. The standard deviation is ≤ 2.2 in all cases.

We noted that in the case of unbound docking the difference between the number of complexes for which a complex structure of a given accuracy is generated (“Top 2000” column in Table 3) and respective “Top 1/10/100” columns is considerably larger than in the case of bound docking (Table 2). This indicates for unbound docking with DrugScore^PPI^/FRODOCK that complex structures of medium and acceptable accuracy can still be generated in many cases but not identified. In comparison to the results from bound docking, this suggests that despite its reduced steepness (see above) DrugScore^PPI^ is still not “soft” enough to compensate for the missing explicit treatment of protein flexibility in the docking algorithm. Structural refinement of complex structures obtained from the docking and re-evaluation with DrugScore^PPI^ could be a way to overcome this limitation [81]. In particular, energy minimization- and/or Monte Carlo-based refinement in internal (dihedral angle) coordinates using a molecular mechanics force field description of the proteins has been successfully applied for this [15], [82]. In addition, two other reasons may give rise to the only fair results. First, DrugScore^PPI^ only evaluates interactions between receptor and ligand atoms that are <5 Å apart. Thus, interactions between atoms potentially being in contact in the native complex will not be considered in the unbound docking if the conformational changes between bound and unbound states are too large. Again, this limitation may be overcome by structural refinement of the initially generated complex structures. Second, the missing long-range interactions in DrugScore^PPI^ may also lead to not recognizing encounter complex configurations, which are generally dominated by electrostatic interactions [83], and can be in equilibrium with, and mutually exclusive to, the specific complex [84]. In fact, when performing the unbound docking with the original FRODOCK implementation, which uses electrostatic interactions for scoring, complex structures of medium and acceptable accuracy were found in the top 10 (top 100) in 22.9% (46.9%) of the cases (Table 3; Table S12 in File S1). This docking accuracy is comparable to that reported in ref. [14]: for a subset of 76 protein-protein complexes obtained by excluding all difficult cases from the ZDOCK benchmark 2.0, an at least acceptable solution was found in the top 20 (top 100) in 30% (51%) of the cases. The docking program ZDOCK version 3.0 considers statistical pair potentials in addition to contributions due to shape, electrostatics, and desolvation [32]. For this docking program, a similar docking accuracy of 25% (50%) for the top 20 (top 100) has been reported on the same 76 protein-protein complexes considering “hits” and “near-hits”, i.e., solutions with an i_rmsd ≤ 4.0 Å similar to the criterion of an at least acceptable accuracy used here [85].

Regarding that critical residues in protein-protein interfaces can be identified efficiently, e.g., by employing alanine scanning [86] or analyzing correlated mutations [87], we probed to what extent such knowledge helps in improving the identification of at least acceptable complex structures with DrugScore^PPI^/FRODOCK. For this we defined a reference point within 5 Å distance of the receptor interface and restricted the space of the translational search for the ligand to 10 Å around this reference point (Figure S3 in File S1). To minimize the bias by the selected reference point on the docking results, each docking run was repeated three times using another randomly selected reference point; in addition, each reference point must be at least 5 Å away from the other two points. Other than that, the same docking parameters were used as for the unbound global docking. An at least acceptable solution is found now in the top 10 (top 100) in 18% (58%) of the cases (Table 3; Table S13 in File S1). This amounts to a ∼2-fold improvement in the success rates compared to the global docking.

**Figure 4:**
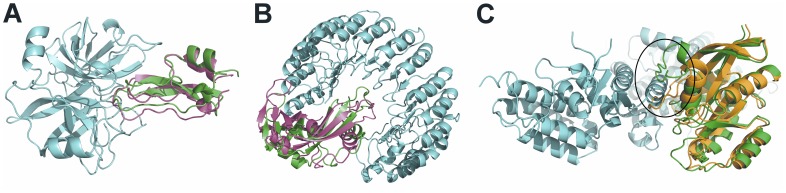
Predictions of unbound protein-protein docking obtained with DrugScore^PPI^/FRODOCK on the top 10 scoring ranks. (A) Medium accuracy complex of MT-SP1/matriptase (cyan) and bovine pancreatic trypsin inhibitor (PDB ID: 1EAW). (B) Acceptable accuracy complex of ribonuclease A (cyan) and a ribonuclease inhibitor. (PDB ID: 1DFJ). In (A) and (B) ligand configurations in the crystal complex are depicted in green, and predicted ligand configurations are colored in magenta. (C) Bound crystal complex of human H-Ras (cyan) and human SOS-1 (green) (PDB ID: 1BKD) onto which the unbound ligand (orange) was aligned. Due to a large conformational change of a loop in the interface (see black ellipse) the generation of a near-native structure failed.

Analyzing the knowledge-driven docking in more detail, the success rate for finding an at least acceptable accuracy solution in the top 10 (top 100) is 20.0% (65.9%) considering only the “easy” and “medium” cases in the benchmark (Figure 4A, B; Table S13 in File S1), i.e., excluding the “difficult” cases where the conformational changes of the binding partners are >2.2 Å Cα-i_rmsd (Figure 4C).

For 13 complexes no solution with i_rmsd <10 Å was found in the top 2000 predictions by knowledge-driven docking with DrugScore^PPI^/FRODOCK, four of which are classified as easy (PDB ID’s: 1I4D, 1I9R, 1SBB, 2VIS), two as medium (1BGX, 1M10), and seven as difficult (1BKD, 1DE4, 1IBR, 1IRA, 1R8S, 1Y64, 2HMI) cases. Four of these complexes are antigen-antibody complexes (PDB-ID’s: 1BGX, 1I9R, 2HMI, 2VIS); one of the complexes has already been discussed above regarding crystal packing effects (PDB-ID: 1I4D). Visual inspection of the remaining eight complexes revealed pronounced conformational changes upon complex formation in terms of loop movements in the interface of five complexes (PDB-ID’s: 1BKD (Figure 4C), 1DE4, 1IBR, 1M10, 1R8S; 2.1<C_α_-i_rmsd<3.7 Å) and large domain movements for one of the complexes (PDB-ID: 1IRA; C_α_-i_rmsd = 8.4 Å). Such large rearrangements in the protein-protein interface are apparently out of the scope of our rigid docking approach using knowledge-based potentials. One way to overcome this limitation without having to modify the rigid docking approach is to perform ensemble docking using ensembles of protein structures deformed along collective degrees of freedom [81], [88], [89], [90]. For the remaining two complexes we could identify issues related to the preparation of the benchmark. First, the complex in PDB-ID 1SBB is given as a one-to-one complex in the benchmark but the biological assembly assigned by the authors in the PDB data base is a dimer where each ligand makes interactions with two receptor molecules. Second, the complex in PDB-ID 1Y64 is also given as a one-to-one complex in the benchmark as found in the asymmetric unit; however, the most likely biologically relevant form is a dimeric FH2 ring (being the receptor) that contacts three successive actin monomers (being the ligands) [91]. Thus, it is not unsurprising that docking these complexes as given in the benchmark fails.

### Estimating when Unbound Protein-protein Docking will be Successful

The success rate of unbound docking with DrugScore^PPI^/FRODOCK is higher for complexes with only small rearrangements (see above). This leads to the question if and how one can estimate *a priori* whether unbound protein-protein docking will be successful; for this, only information on the unbound binding partners should be used so as to mimic a real-life scenario. To this end, we applied a method developed by Marsh *et al.* [92] that uses the relative solvent accessible surface area (A_rel_) of an unbound protein in order to predict the magnitude of binding-induced conformational changes. A_rel_ is the actual accessible surface area of a protein divided by the accessible surface area expected for a folded protein of the same molecular weight. Using this measure follows the rationale that binding partners in the unbound state with higher A_rel_ values expose more surface area and adopt more extended conformations, thus, they are likely to be more flexible and, hence, show larger conformational changes upon binding [92]. Indeed Marsh *et al.* found a linear correlation between A_rel_ and log(rmsd between the bound and unbound state) with *r* ^2^ = 0.64. When computing A_rel_ for those 88 binding partners in the cleaned version of the ZDOCK benchmark 3.0 where both of the proteins are in the unbound state, we find a linear correlation of *r* ^2^ = 0.49 (*p*<0.001) with the logarithm of the all-atom rmsd value with respect to the bound conformation (Figure S4 in File S1). The same correlation is obtained if the C_α_ atom rmsd is used instead. The difference between our results and those from ref. [92] may reflect a dataset dependence. Note that our dataset set only contains heteromers because Marsh *et al.* stated that the A_rel_ vs. log(rmsd) correlation is weak for homomers [92].

Relating A_rel_ to the results from our knowledge-driven unbound protein-protein docking, we find that for 80% of the complexes where the docking failed at least one protein had A_rel_ >1.1 (Table S14 & Figure S4 in File S1). Along the same lines, if at least one protein has A_rel_ >1.1, the likelihood to get an at least acceptable accuracy solution in the top 100 is 48% only (Table S14 in File S1). In contrast, if both of the proteins have A_rel_ <1.1, the likelihood to get an at least acceptable accuracy solution in the top 100 is 85.4% (Table S14 in File S1). Considering this as a binary classification problem, the A_rel_ criterion discriminates between successful unbound dockings and unsuccessful ones with a sensitivity of 85%, a specificity of 100%, and an accuracy of 92% (Figure S5 in File S1). Thus, using the simple measure A_rel_ is a valuable means for predicting when unbound protein-protein docking of heteromeric structures with DrugScore^PPI^/FRODOCK will be successful.

### Concluding Remarks

In summary, the distance-dependent knowledge-based DrugScore^PPI^ potentials have been evaluated as a scoring and objective function in structure prediction of protein-protein complexes. When applied for ranking “unbound perturbation” and “unbound docking” decoys generated by Baker and coworkers, DrugScore^PPI^ results in a 4-fold enrichment of acceptable docking solutions in the top ranks compared to a random selection in the former case, and a 1.5-fold enrichment (with respect to the R5Å criterion) in the latter case. Compared to the results by Baker and coworkers, DrugScore^PPI^ performs slightly inferior in the case of the “unbound perturbation” dataset but superior in the case of the “unbound docking” dataset. When applied as an objective function in FRODOCK for bound protein-protein docking on 97 complexes of the ZDOCK benchmark 3.0, DrugScore^PPI^/FRODOCK finds up to 10% (15%) more high accuracy solutions in the top 1 (top 10) predictions than the original FRODOCK implementation. In contrast, when used as an objective function for global unbound protein-protein docking, only fair docking success rates are obtained. They improve by ∼2-fold to 18% (58%) for an at least acceptable solution in the top 10 (top 100) predictions when performing knowledge-driven unbound docking. These docking success rates are comparable to those of other state-of-the-art protein-protein docking approaches. Finally, we devised a highly accurate criterion based on the relative solvent accessible surface area (A_rel_) for *a priori* prediction when unbound protein-protein docking of heteromeric structures with DrugScore^PPI^/FRODOCK will be successful.

Our results are remarkable as DrugScore^PPI^ has been originally developed for *in silico* alanine scanning and hot spot prediction on given structures of protein-protein complexes. So far, no tweaking of parameters with respect to evaluating protein-protein complex structures has been done, in contrast to optimization procedures applied to other scoring functions for protein-protein docking [14], [24], [85], [93]. This indicates that DrugScore^PPI^ already balances very well several different types of (short-range) interactions important for protein-protein recognition. Our analysis of the unbound docking results suggests that augmenting DrugScore^PPI^ by additional (long-range) terms, as done in other studies [32], [33], should further improve its power for the structure prediction of protein-protein complexes.

## Supporting Information

File S1 The file *DSPPI_PPD_suppinfo_final_correct.pdf* contains additional information to the manuscript explaining datasets, methods, and results in further details. It consists of 36 pages, 14 tables and 5 figures.
(PDF)
