# Supplementary material for: DrugScorePPI Knowledge-Based Potentials Used as Scoring and Objective Function in Protein-Protein Docking
Source: PLoS One. 2014 Feb 21;9(2):e89466. doi: 10.1371/journal.pone.0089466 (PMC3931789; doi:10.1371/journal.pone.0089466)
Supplement: File S1 — The file DSPPI_PPD_suppinfo_final_correct.pdf contains additional information to the manuscript explaining datasets, methods, and results in further details. It consists of 36 pages, 14 tables and 5 figures. (PDF) [file pone.0089466.s001.pdf]

Supporting Information

DrugScore^{PPI} Knowledge-Based Potentials Used as Scoring and Objective Function in Protein-Protein Docking

Dennis M. Krüger[1], José Ignacio Garzón[2], Pablo Chacón[2], Holger Gohlke[1*]

[1]Institute for Pharmaceutical and Medicinal Chemistry, Heinrich-Heine-University, Düsseldorf, Germany

[2]Rocasolano Physical Chemistry Institute, Consejo Superior de Investigaciones Científicas, Madrid, Spain

[*]Universitätsstr.1, 40225 Düsseldorf (Germany), Fax: (+49) 211-8113847, E-mail: gohlke@uni-duesseldorf.de

Supplemental Tables

Table S1: 27 targets skipped from the ZDOCK benchmark 3.0 [1].

PDB ID	Difficulty	Category[a]
1A2K	Easy	O
1ACB	Medium	E
1ATN	Difficult	O
1AZS	Easy	O
1E4K	Difficult	A
1E6E	Easy	E
1E96	Easy	O
1EFN	Easy	O
1F34	Easy	E
1FAK	Difficult	O
1FC2	Easy	O
1FQ1	Difficult	E
1GHQ	Easy	O
1GP2	Medium	O
1GRN	Medium	O
1H1V	Difficult	O
1HE8	Medium	O
1IB1	Medium	O
1IJK	Medium	E
1J2J	Easy	O
1JMO	Difficult	O
1KLU	Easy	O
1NW9	Medium	E
1Z0K	Easy	O
1Z5Y	Easy	O
2O8V	Easy	E
2OOB	Easy	O

[a] Complex category labels: E = Enzyme/inhibitor or enzyme/substrate, A = Antigen-antibody, AB = Antigen-bound antibody, O = Others.

Table S2: Computational efficiency of docking with DrugScore^{PPI}/FRODOCK and the original FRODOCK implementation.[a]

Runtime [b]		PDB ID	no. of atoms			max. diameter [c]
**DrugScore^{PPI}/ FRODOCK**	**Original FRODOCK**		**complex**	**receptor**	**ligand**	
18	15	1ACB	2291	1768	522	48
23	9	2PCC	3218	2371	847	55
330	130	1AHW	4916	3304	1612	76

[a] Using 16 cores on dual CPU compute servers equipped with 2 GHz Intel Xeon Quadcore CPUs, 24 GB of RAM, and Infiniband interconnect.

[b] In min.

[c] Maximum diameter of the receptor, which influences the size of the search space. In Å.

Table S3: DrugScore[PPI] potential maps and corresponding Sybyl atomtypes.[a]

Potential map	Considered atomtype(s)[a]	Definition
C_2	C.2	sp^2 carbon
C_3	C.3	sp^3 carbon
C_ar	C.ar	aromatic carbon
C_cat	C.cat	carbocation (C+) in a guanidinium group
N_3	N.2, N.3, N.4	nitrogen sp^2, sp^3, sp^3 positively charged
N_am	N.am	amide nitrogen
N_pl3	N.pl3	trigonal planar nitrogen
O_2	O.2	sp^2 oxygen
O_3	O.3	sp^3 oxygen
O_co2	O.co2	oxygen in a carboxylate group
S_3	S.3	sp^3 sulfur

[a] Sybyl atomtype notation from Tripos, available at http://tripos.com/mol2/atom_types.html.

Table S4: Distribution of the decoy quality in the “unbound perturbation” dataset.

PDB ID[a]	< 2 Å [b]	< 5 Å [b]	< 10 Å [b]	≥ 10 Å [b]
1A0O	0.00	0.60	27.76	72.24
1ACB	0.10	6.00	25.70	74.30
1AHW	0.00	1.23	14.93	85.07
1ATN	4.60	9.91	28.60	71.40
1AVW	0.00	5.44	29.91	70.09
1AVZ	0.00	0.70	15.72	84.28
1BQL	0.70	5.73	20.32	79.68
1BRC	1.00	5.00	29.60	70.40
1CGI	0.40	8.21	27.63	72.37
1BRS	0.10	4.30	35.14	64.86
1CHO	0.10	6.30	30.50	69.50
1BTH	0.00	0.00	17.90	82.10
1BVK	0.00	1.31	23.84	76.16
1DFJ	0.00	2.42	20.14	79.86
1CSE	0.00	8.08	33.23	66.77
1DQJ	0.00	0.30	16.28	83.72
1EFU	0.00	0.20	6.53	93.47
1FBI	0.00	6.23	25.83	74.17
1EO8	0.00	0.60	15.88	84.12
1FIN	0.00	0.00	5.23	94.77
1FQ1	0.00	0.60	22.61	77.39
1FSS	0.40	14.70	31.50	68.50
1GLA	0.10	4.20	32.23	67.77
1GOT	0.00	0.20	9.18	90.82
1IAI	0.00	4.00	15.40	84.60
1IGC	0.70	2.30	31.30	68.70
1JHL	0.00	1.70	27.40	72.60
1MAH	0.71	21.01	37.17	62.83
1MDA	0.61	8.99	30.10	69.90
1MEL	0.00	5.04	35.75	64.25
1MLC	0.40	7.16	28.15	71.85
1NCA	1.03	10.70	30.66	69.34
2BTF	0.61	9.11	29.96	70.04
1NMB	6.28	19.13	33.20	66.80
1PPE	4.92	17.47	48.59	51.41
1QFU	0.30	9.50	28.01	71.99
1TAB	0.10	16.78	34.87	65.13
1SPB	5.91	13.21	32.53	67.47
1TGS	2.02	6.98	30.16	69.84
1STF	12.63	21.44	32.57	67.43
1UDI	0.80	20.26	42.23	57.77
2JEL	0.20	6.90	42.50	57.50
1UGH	3.61	17.23	38.98	61.02
2KAI	0.80	19.90	41.00	59.00
1WEJ	0.00	2.22	24.65	75.35
1WQ1	0.00	5.20	34.15	65.85
2PCC	0.00	1.91	28.74	71.26
2PTC	1.61	5.83	29.05	70.95

3HHR	0.00	0.20	10.61	89.39
2SIC	0.50	17.39	33.67	66.33
2SNI	0.40	9.85	29.95	70.05
2TEC	4.21	13.04	31.90	68.10
2VIR	0.70	6.32	37.81	62.19
4HTC	0.30	10.91	28.13	71.87
**Average**	**1.05**	**7.48**	**27.88**	**72.12**

[a] Dataset from Baker and coworkers [2].
[b] Percentage of decoys that are within the given all-atom rmsd range.

Table S5: Distribution of the decoy quality in the “unbound docking” dataset.[a]

PDB ID[b]	< 2 Å [c]	< 5 Å [c]	< 10 Å [c]	≥ 10 Å [c]
1A0O	0.00	0.50	19.00	81.00
1ACB	*0.00*	*0.50*	*3.00*	*97.00*
1AHW	0.00	0.00	5.50	94.50
1ATN	2.50	4.50	7.00	93.00
1AVW	0.00	0.50	7.50	92.50
1AVZ	*0.00*	*0.00*	*1.00*	*99.00*
1BQL	2.50	10.50	17.50	82.50
1BRC	*0.00*	*0.00*	*0.00*	*100.00*
1CGI	0.50	10.00	20.50	79.50
1BRS	0.00	1.00	15.00	85.00
1CHO	0.00	3.00	20.00	80.00
1BTH	*0.00*	*0.00*	*0.00*	*100.00*
1BVK	0.00	0.00	75.70	24.30
1DFJ	*0.00*	*0.00*	*0.00*	*100.00*
1CSE	0.00	0.00	9.50	90.50
1DQJ	*0.00*	*0.00*	*1.00*	*99.00*
1EFU	*0.00*	*0.00*	*0.50*	*99.50*
1FBI	*0.00*	*1.50*	*1.50*	*98.50*
1EO8	*0.00*	*0.00*	*0.00*	*100.00*
1FIN	*0.00*	*0.00*	*0.00*	*100.00*
1FQ1	*0.00*	*0.00*	*1.00*	*99.00*
1FSS	0.00	3.00	3.50	96.50
1GLA	*0.00*	*0.00*	*0.00*	*100.00*
1GOT	*0.00*	*0.00*	*0.00*	*100.00*
1IAI	*0.00*	*0.00*	*0.00*	*100.00*
1IGC	*0.00*	*0.00*	*0.00*	*100.00*
1JHL	0.00	0.00	6.12	93.88
1MAH	0.50	7.00	7.50	92.50
1MDA	*0.00*	*0.00*	*0.00*	*100.00*
1MEL	0.00	5.00	20.00	80.00
1MLC	0.50	3.50	4.50	95.50
1NCA	*0.00*	*0.00*	*0.00*	*100.00*
2BTF	*0.00*	*0.00*	*0.00*	*100.00*
1NMB	*0.00*	*0.00*	*0.00*	*100.00*
1PPE	7.50	24.50	83.00	17.00
1QFU	*0.00*	*1.00*	*1.50*	*98.50*
1TAB	0.00	12.50	22.50	77.50
1SPB	3.00	4.50	7.50	92.50
1TGS	0.50	6.50	25.50	74.50
1STF	4.50	8.00	9.00	91.00
1UDI	0.00	5.35	10.16	89.84
2JEL	2.50	16.50	41.50	58.50
1UGH	3.00	18.00	35.50	64.50
2KAI	1.50	30.50	41.50	58.50
1WEJ	*0.00*	*0.00*	*0.00*	*100.00*
1WQ1	0.00	0.00	4.00	96.00
2PCC	*0.00*	*0.00*	*0.00*	*100.00*
2PTC	0.50	2.00	3.50	96.50

3HHR	*0.00*	*0.00*	*0.00*	*100.00*
2SIC	0.00	7.50	9.00	91.00
2SNI	1.00	3.00	10.00	90.00
2TEC	2.50	8.00	11.50	88.50
2VIR	*0.00*	*0.00*	*0.00*	*100.00*
4HTC	0.00	5.00	20.00	80.00
**Average[d]**	**1.10 (0.00)**	**6.68 (0.13)**	**19.10 (0.40)**	**80.90 (99.60)**

[a] 24 targets where 97% or more of the decoys have an all-atom rmsd > 10Å are marked in grey. For 17 out of these 24 decoys no solution with an all-atom rmsd < 10Å was generated. For 7 decoys only one solution with an all-atom rmsd < 10Å was found in the whole decoy set.
[b] Dataset from Baker and coworkers [2].
[c] Percentage of decoys that are within the given all-atom rmsd range.
[d] Numbers not in brackets refer to decoy sets where at least two decoys with an all-atom rmsd < 10Å are available; numbers in brackets consider decoy sets where less than two decoys with rmsd < 10Å are available.

Table S6: Results from rescoring "unbound perturbation" decoys with DrugScore^PPI.

PDB ID[a]	N10Å[b]		Best rmsd[c]	
	This work	Baker and coworkers	This work	Baker and coworkers
1A0O	1	1	8.35	8.22
1ACB	0	2	11.14	9.08
1AHW	3	5	7.40	6.17
1ATN	5	5	2.37	0.96
1AVW	5	5	5.14	6.11
1AVZ	0	0	10.12	10.14
1BQL	1	5	1.57	1.40
1BRC	4	1	3.60	3.76
1BRS	6	4	8.42	3.89
1BTH	6	0	5.15	18.23
1BVK	3	5	6.70	6.25
1CGI	6	4	3.24	3.15
1CHO	1	3	5.25	5.61
1CSE	4	2	6.11	8.65
1DFJ	6	4	4.30	3.92
1DQJ	3	2	6.65	5.86
1EFU	0	0	19.31	10.64
1EO8	0	1	12.35	7.38
1FBI	2	3	3.58	2.71
1FIN	0	0	14.82	16.56
1FQ1	4	2	7.34	9.61
1FSS	6	5	4.48	2.98
1GLA	0	1	15.17	5.95
1GOT	0	0	16.60	19.27
1IAI	4	0	3.96	14.11
1IGC	4	2	1.92	1.92
1JHL	3	1	6.11	8.56
1MAH	6	5	4.60	2.36
1MDA	2	3	3.00	7.86
1MEL	4	5	4.43	5.38
1MLC	0	0	11.16	18.89
1NCA	6	5	1.24	1.24
1NMB	1	5	2.66	0.65
1PPE	6	5	1.38	0.61
1QFU	0	5	14.28	2.00
1SPB	6	5	0.88	0.91
1STF	5	5	1.57	1.31
1TAB	4	5	3.37	3.67
1TGS	5	5	0.62	1.39
1UDI	5	5	2.08	1.16
1UGH	6	5	1.68	1.69
1WEJ	4	0	6.83	10.65
1WQ1	2	3	5.81	3.99
2BTF	2	4	2.40	1.90
2JEL	6	5	4.71	4.71
2KAI	6	4	1.21	2.04
2PCC	4	3	8.76	7.28

2PTC	4	2	5.20	0.98
2SIC	6	5	2.64	2.41
2SNI	6	4	1.61	1.61
2TEC	5	5	1.97	2.25
2VIR	3	4	4.99	6.12
3HHR	0	0	12.53	10.75
4HTC	6	5	3.64	3.81
**Totals**	**31**[d]	**34** [d]	**44**[e]	**45**[e]

[a] Dataset from Baker and coworkers [2].
[b] No of decoys that have all-atom rmsd < 10Å in the top 5.
[c] Best all-atom rmsd in the top 5. In Å.
[d] Totals count the number of targets that have at least three solutions with an all-atom rmsd < 10Å in the top 5.
[e] Totals count the number of targets that have an all-atom rmsd < 10Å in the top 5.

Table S7: Results from rescoring "unbound docking" decoys with DrugScore^PPI.

PDB ID[a]	Best rmsd[b]	
	This work	Baker and coworkers
1A0O	5.08	5.08
1ACB	12.29	11.84
1AHW	7.57	7.24
1ATN	2.3	2.30
1AVW	6.47	5.76
1AVZ	19.84	20.03
1BQL	1.24	2.09
1BRC	15.96	15.96
1BRS	6.59	8.67
1BTH	17.37	16.96
1BVK	6.32	6.15
1CGI	3.88	3.27
1CHO	4.82	6.88
1CSE	8.59	8.87
1DFJ	20.59	22.09
1DQJ	17.75	17.75
1EFU	29.51	29.51
1EO8	29.43	56.51
1FBI	20.99	22.25
1FIN	27.15	26.84
1FQ1	25.59	24.64
1FSS	2.34	3.19
1GLA	53.04	25.66
1GOT	55.46	55.84
1IAI	34.04	33.96
1IGC	28.09	24.19
1JHL	9.4	14.91
1MAH	2.4	2.40
1MDA	54.18	53.71
1MEL	3.56	5.80
1MLC	6.52	2.52
1NCA	31.28	31.47
1NMB	72.82	72.48
1PPE	3.66	0.59
1QFU	28.55	3.20
1SPB	0.9	1.32
1STF	2.32	0.56
1TAB	3.78	3.35
1TGS	4.48	3.31
1UDI	3.75	3.75
1UGH	1.28	2.15
1WEJ	17.9	17.90
1WQ1	5.08	44.03
2BTF	11.97	27.52
2JEL	3.42	1.45
2KAI	3.09	2.40
2PCC	18.75	19.07

2PTC	9.69	10.35
2SIC	4.93	2.98
2SNI	1.36	1.36
2TEC	0.87	2.09
2VIR	56.65	41.47
3HHR	33.95	33.90
4HTC	4.8	3.84
**R10Å[c]**	**24 (6)**	**22 (6)**
**R5Å[d]**	**17 (5)**	**15 (5)**

[a] Dataset from Baker and coworkers [2].
[b] Best all-atom rmsd in the top 10. In Å. Decoys were clustered according to Baker and coworkers [2].
[c] Number of targets that have at least one solution with all-atom rmsd < 10Å in the first solutions of the top 10 clusters. Numbers not in brackets refer to decoy sets where at least two decoys with rmsd < 10Å are available; numbers in brackets refer to decoy sets where less than two decoys with rmsd < 10Å are available.
[d] Number of targets that have at least one solution with all-atom rmsd < 5Å in the first solutions of the top 10 clusters. Numbers not in brackets refer to decoy sets where at least two decoys with rmsd < 10Å are available; numbers in brackets refer to decoy sets where less than two decoys with rmsd < 10Å are available.

Table S8: Protein-protein complexes where multiple ligand binding modes have to be considered for rmsd calculations.

Difficulty	Category[a]	PDB ID
Easy	E	1EZU
Easy	O	1F51
Easy	AB	1I9R
Easy	AB	1QFW
Easy	O	1RLB
Easy	E	2PCC
Easy	O	1SBB
Medium	E	1KKL
Medium	O	1N2C
Medium	O	2H7V
Difficult	O	1DE4

[a] Complex category labels: E = Enzyme/inhibitor or enzyme/substrate, A = Antigen-antibody, AB = Antigen-bound antibody, O = Others.

Table S9: Results for bound docking with the DrugScore^{PPI}/FRODOCK approach.[a]

Difficulty	Category[b]	PDB ID[c]	Quality_1[d]	Quality_2[e]	Quality_3[f]	i_rmsd[g]	l_rmsd[h]	f_{nat}[i]	f_{not}[j]
Easy	A	1AHW	-	260	260	1.58	2.19	0.92	0.25
Easy	O	1AK4	-	91	91	1.43	3.73	0.83	0.18
Easy	O	1AKJ	-	21	21	2.11	4.34	0.85	0.32
Easy	E	1AVX	-	1	1	1.27	2.96	0.94	0.26
Easy	E	1AY7	-	1	1	1.09	1.31	0.96	0.16
Easy	O	1B6C	-	1	1	1.53	1.63	0.97	0.25
Medium	A	1BGX	-	1	1	2.45	4.72	0.82	0.26
Easy	AB	1BJ1	4	4	4	0.84	2.02	0.93	0.05
Difficult	O	1BKD	1	1	1	0.48	0.57	0.92	0.03
Easy	O	1BUH	-	7	7	1.12	1.06	0.83	0.04
Easy	A	1BVK	10	10	10	0.97	1.19	0.92	0.18
Easy	E	1BVN	1	1	1	0.39	0.41	0.96	0.12
Easy	E	1CGI	1	1	1	0.92	0.94	0.82	0.03
Easy	E	1D6R	1	1	1	0.62	0.56	0.94	0.03
Difficult	O	1DE4	-	803	803	1.55	2.50	0.97	0.27
Easy	E	1DFJ	-	1	1	1.44	1.70	0.86	0.24
Easy	A	1DQJ	1	1	1	1.03	1.06	0.97	0.27
Easy	A	1E6J	-	-	-	-	-	-	-
Easy	E	1EAW	-	1	1	1.18	1.39	0.95	0.14
Difficult	O	1EER	1	1	1	0.72	0.66	0.90	0.03
Easy	E	1EWY	-	943	2	9.60	9.68	0.18	0.89
Easy	E	1EZU	1	1	1	0.72	0.59	0.93	0.08
Easy	O	1F51	-	1	1	1.31	1.08	0.85	0.15
Easy	O	1FQJ	-	3	3	1.20	1.50	0.88	0.11
Easy	AB	1FSK	-	1	1	1.09	1.15	0.93	0.18
Easy	O	1GCQ	-	1	1	1.59	1.52	0.93	0.23
Easy	O	1GLA	-	-	-	-	-	-	-
Easy	O	1GPW	-	1	1	1.56	2.12	0.85	0.23
Easy	O	1HE1	1	1	1	0.59	0.69	0.89	0.02
Easy	E	1HIA	1	1	1	0.74	1.07	0.91	0.14
Medium	O	1I2M	-	1	1	1.16	1.16	0.91	0.18
Easy	O	1I4D	-	-	-	-	-	-	-
Easy	AB	1I9R	-	-	-	-	-	-	-
Difficult	O	1IBR	-	1	1	1.08	1.08	0.91	0.10
Easy	AB	1IQD	-	1	1	1.15	1.16	0.85	0.15
Difficult	O	1IRA	-	1	1	1.41	1.44	0.87	0.27
Easy	A	1JPS	-	8	8	1.57	1.55	0.95	0.34
Easy	AB	1K4C	-	2	2	1.09	1.06	0.90	0.15
Medium	O	1K5D	-	9	9	1.61	2.14	0.83	0.16
Easy	O	1K74	-	2	2	1.51	2.78	0.82	0.19
Easy	O	1KAC	-	184	81	7.17	9.56	0.31	0.78
Medium	E	1KKL	-	12	12	1.21	2.04	0.89	0.19
Easy	O	1KTZ	-	491	491	3.78	6.81	0.69	0.62
Easy	O	1KXP	-	3	3	1.81	3.21	0.80	0.13
Easy	AB	1KXQ	-	1	1	1.13	1.13	0.95	0.25
Medium	E	1M10	-	2	2	2.12	3.24	0.80	0.12
Easy	E	1MAH	-	1	1	1.72	2.20	0.86	0.24

Easy	O	1ML0	-	2	2	1.39	1.34	0.96	0.23
Easy	A	1MLC	18	18	18	1.00	1.09	0.93	0.16
Medium	O	1N2C	1	1	1	0.95	0.95	0.88	0.23
Easy	E	1N8O	-	1	1	1.50	3.10	0.92	0.15
Easy	AB	1NCA	-	3	3	2.43	4.25	0.75	0.19
Easy	AB	1NSN	-	250	246	8.07	9.97	0.31	0.80
Easy	E	1OPH	-	4	4	1.95	3.45	0.91	0.18
Easy	E	1PPE	-	1	1	1.99	1.95	0.89	0.31
Difficult	E	1PXV	-	1	1	1.61	2.79	0.88	0.19
Easy	O	1QA9	-	988	469	9.24	9.85	0.21	0.83
Easy	AB	1QFW	-	18	18	1.45	1.29	0.93	0.21
Easy	E	1R0R	-	1	1	1.57	4.96	0.92	0.24
Difficult	O	1R8S	-	1	1	3.75	5.60	0.78	0.27
Easy	O	1RLB	-	-	609	7.71	9.83	0.30	0.76
Easy	O	1S1Q	28	28	28	0.96	0.94	0.94	0.19
Easy	O	1SBB	-	-	-	-	-	-	-
Easy	O	1T6B	-	3	3	1.77	2.76	0.85	0.24
Easy	E	1TMQ	-	1	1	1.75	1.73	0.97	0.38
Easy	E	1UDI	-	1	1	1.43	1.74	0.95	0.22
Easy	A	1VFB	9	9	9	0.72	0.88	0.88	0.06
Easy	A	1WEJ	312	15	15	1.80	3.72	0.79	0.22
Medium	O	1WQ1	1	1	1	0.92	1.44	0.91	0.08
Easy	O	1XD3	-	1	1	5.71	5.47	0.91	0.16
Medium	O	1XQS	-	1	1	1.84	4.35	0.79	0.17
Difficult	O	1Y64	-	40	40	1.50	2.68	0.88	0.32
Easy	E	1YVB	2	1	1	2.25	5.96	0.75	0.35
Easy	O	1ZHI	-	65	65	1.15	1.18	0.96	0.29
Easy	O	2AJF	-	644	155	6.81	8.03	0.47	0.71
Easy	E	2B42	-	1	1	1.11	1.48	0.96	0.24
Easy	O	2BTF	-	1	1	1.30	1.45	0.71	0.06
Difficult	O	2C0L	-	1	1	2.43	4.14	0.95	0.27
Medium	O	2CFH	-	1	1	1.67	1.91	0.93	0.31
Easy	A	2FD6	-	666	55	8.93	9.54	0.25	0.82
Medium	O	2H7V	-	79	79	1.08	1.37	0.86	0.06
Easy	O	2HLE	1	1	1	0.85	5.50	0.94	0.12
Difficult	AB	2HMI	-	-	-	-	-	-	-
Easy	O	2HQS	-	1	1	1.51	3.41	0.97	0.20
Medium	O	2HRK	6	6	6	0.89	1.00	0.92	0.02
Easy	A	2I25	1	1	1	0.67	0.94	0.95	0.03
Easy	AB	2JEL	-	1	1	1.30	1.80	0.85	0.10
Easy	E	2MTA	-	105	105	1.66	2.55	0.88	0.38
Medium	O	2NZ8	-	1	1	1.23	1.18	0.93	0.22
Difficult	O	2OT3	-	1	1	1.12	1.12	0.94	0.09
Easy	E	2PCC	-	-	226	6.93	9.83	0.44	0.73
Easy	AB	2QFW	1	1	1	0.98	1.13	0.96	0.15
Easy	E	2SIC	-	1	1	1.96	2.48	0.83	0.24
Easy	E	2SNI	-	1	1	1.09	6.01	0.92	0.28
Easy	E	2UUY	-	1944	1944	3.00	9.00	0.77	0.35
Easy	A	2VIS	1	1	1	1.05	1.58	0.91	0.10
Easy	E	7CEI	-	260	260	1.58	2.19	0.92	0.25

[a] Subset of 97 structures from the ZDOCK benchmark 3.0.
[b] Complex category labels: E = Enzyme/inhibitor or enzyme/substrate, A = Antigen-antibody, AB = Antigen-bound antibody, O = Others.
[c] Subset of 27 structures skipped from the ZDOCK benchmark 3.0. For details see Materials and Methods section.
[d] Rank of the first solution that has a high accuracy. The lower the rank, the better is the prediction. “-“ denotes that no solution was found.
[e] Rank of the first solution that has at least medium accuracy. The lower the rank, the better is the prediction. “-“ denotes that no solution was found.
[f] Rank of the first solution that has at least acceptable accuracy. The lower the rank, the better is the prediction. “-“ denotes that no solution was found. Additional details for this case are given in the subsequent columns.
[g]: All-atom interface RMSD of the docked ligand and its bound crystal structure conformation.
[h]: All-atom RMSD of the docked ligand and its bound crystal structure conformation.
[i]: Fraction of native residue-residue contacts of the docked ligand conformation compared to its bound crystal structure conformation.
[j]: Fraction of non-native residue-residue contacts of the docked ligand conformation compared to its bound crystal structure conformation.

Table S10: Results for bound docking with the original FRODOCK implementation.[a]

Difficulty	Category	PDB ID	Quality_1	Quality_2	Quality_3	i_rmsd	l_rmsd	f_{nat}	f_{not}
Easy	A	1AHW	-	10	10	1.71	1.89	0.82	0.19
Easy	O	1AK4	-	546	546	7.47	9.54	0.73	0.45
Easy	O	1AKJ	-	665	665	3.48	5.61	0.68	0.37
Easy	E	1AVX	-	1	1	2.12	3.56	0.97	0.26
Easy	E	1AY7	-	1	1	1.63	2.19	0.88	0.18
Easy	O	1B6C	-	1	1	3.16	3.73	0.89	0.25
Medium	A	1BGX	-	1	1	2.02	2.16	0.82	0.25
Easy	AB	1BJ1	-	6	6	1.29	2.95	0.83	0.13
Difficult	O	1BKD	1	1	1	0.88	0.97	0.85	0.08
Easy	O	1BUH	27	27	27	0.81	0.87	0.94	0
Easy	A	1BVK	-	24	24	2.11	2.46	0.69	0.08
Easy	E	1BVN	-	1	1	2.99	3.36	0.78	0.23
Easy	E	1CGI	1	1	1	0.91	0.94	0.87	0.03
Easy	E	1D6R	-	27	27	1.91	2.95	0.88	0.1
Difficult	O	1DE4	23	23	23	0.89	1.14	0.91	0.04
Easy	E	1DFJ	-	329	52	6.32	6.41	0.45	0.77
Easy	A	1DQJ	-	12	12	1.24	1.54	0.85	0.15
Easy	A	1E6J	-	-	-	-	-	-	-
Easy	E	1EAW	-	1	1	2.09	2.15	0.91	0.35
Difficult	O	1EER	-	1	1	2.01	2.52	0.82	0.22
Easy	E	1EWY	-	3	3	2.96	2.91	0.83	0.5
Easy	E	1EZU	-	2	2	2.02	2.55	0.8	0.17
Easy	O	1F51	-	4	4	1.59	1.87	0.89	0.11
Easy	O	1FQJ	-	119	74	5.21	6.64	0.44	0.55
Easy	AB	1FSK	-	1	1	1.95	2.88	0.72	0.23
Easy	O	1GCQ	-	20	20	1.67	2.03	0.9	0.21
Easy	O	1GLA	-	89	89	5.1	6	0.6	0.38
Easy	O	1GPW	-	1	1	3.59	5.9	0.71	0.55
Easy	O	1HE1	-	1	1	1.75	1.63	0.65	0.08
Easy	E	1HIA	-	1	1	1.35	1.42	0.94	0.21
Medium	O	1I2M	-	1	1	1.72	1.89	0.77	0.09
Easy	O	1I4D	-	-	-	-	-	-	-
Easy	AB	1I9R	-	-	-	-	-	-	-
Difficult	O	1IBR	-	1	1	1.56	1.82	0.91	0.15
Easy	AB	1IQD	-	6	6	1.56	2.34	0.89	0.11
Difficult	O	1IRA	-	1	1	2.17	2.38	0.78	0.31
Easy	A	1JPS	-	11	11	2.41	3	0.9	0.27
Easy	AB	1K4C	-	499	499	1.35	2.31	0.81	0.09
Medium	O	1K5D	1	1	1	1.05	1.09	0.91	0.09
Easy	O	1K74	-	3	3	5.27	9.31	0.71	0.55
Easy	O	1KAC	51	20	20	2.68	4.6	0.8	0.18
Medium	E	1KKL	16	16	16	0.98	1.08	0.95	0.08
Easy	O	1KTZ	-	158	158	2.32	3.19	0.9	0.38
Easy	O	1KXP	-	1	1	1.59	2.65	0.86	0.12
Easy	AB	1KXQ	-	1	1	2.01	2.7	0.89	0.25
Medium	E	1M10	-	1	1	2.01	2.55	0.93	0.19
Easy	E	1MAH	-	1	1	1.89	1.93	0.92	0.26
Easy	O	1ML0	-	2	2	2.31	2.54	0.85	0.2

Easy	A	1MLC	-	4	4	1.99	3.32	0.82	0.25
Medium	O	1N2C	-	3	3	2.26	3.14	0.71	0.08
Easy	E	1N8O	-	1	1	2.14	3.14	0.91	0.16
Easy	AB	1NCA	-	28	28	1.32	2.45	0.82	0.11
Easy	AB	1NSN	-	160	160	1.87	1.95	0.78	0.28
Easy	E	1OPH	-	51	51	2.56	3.48	0.87	0.11
Easy	E	1PPE	1	1	1	0.82	0.83	0.96	0.15
Difficult	E	1PXV	-	1	1	1.37	1.76	0.9	0.11
Easy	O	1QA9	-	-	-	-	-	-	-
Easy	AB	1QFW	-	186	186	1.68	2.77	0.92	0.24
Easy	E	1R0R	-	2	2	4.49	4.88	0.93	0.14
Difficult	O	1R8S	-	6	4	7.43	8.11	0.43	0.37
Easy	O	1RLB	-	728	208	7.06	8.83	0	1
Easy	O	1S1Q	-	153	153	4.27	5.06	0.63	0.45
Easy	O	1SBB	-	-	-	-	-	-	-
Easy	O	1T6B	-	26	26	1.98	2.61	0.96	0.34
Easy	E	1TMQ	-	1	1	1.52	1.55	0.98	0.29
Easy	E	1UDI	-	1	1	1.56	1.75	0.83	0.06
Easy	A	1VFB	-	24	24	1.63	2.08	0.75	0.05
Easy	A	1WEJ	-	80	80	3.32	5.1	0.82	0.29
Medium	O	1WQ1	-	-	36	5.12	6.05	0.4	0.5
Easy	O	1XD3	-	1	1	6.25	5.43	0.88	0.09
Medium	O	1XQS	-	20	1	1.07	1.89	0	1
Difficult	O	1Y64	-	2	2	2.91	4.37	0.78	0.52
Easy	E	1YVB	-	1	1	3.76	6.6	0.63	0.49
Easy	O	1ZHI	117	62	62	3.57	5.72	0.93	0.36
Easy	O	2AJF	-	55	55	1.14	1.46	0.85	0.18
Easy	E	2B42	-	1	1	2.84	3.66	0.78	0.22
Easy	O	2BTF	1	1	1	0.86	0.9	0.84	0.01
Difficult	O	2C0L	-	2	2	3.17	4.66	0.95	0.33
Medium	O	2CFH	-	1	1	2.01	2.68	0.9	0.26
Easy	A	2FD6	-	161	2	7.88	8.51	0.15	0.9
Medium	O	2H7V	-	82	82	1.5	2.76	0.89	0.11
Easy	O	2HLE	-	1	1	3.08	6.76	0.78	0.11
Difficult	AB	2HMI	-	1575	1575	1.79	3.52	0.91	0.07
Easy	O	2HQS	-	1	1	4.07	4.26	0.81	0.22
Medium	O	2HRK	-	19	19	1.58	1.7	0.85	0.05
Easy	A	2I25	-	1	1	1.43	1.59	0.89	0.04
Easy	AB	2JEL	32	32	32	0.88	1.02	0.91	0.12
Easy	E	2MTA	-	2	2	1.39	2.37	0.94	0.23
Medium	O	2NZ8	-	1	1	1.78	1.99	0.95	0.29
Difficult	O	2OT3	-	1	1	1.68	1.91	0.78	0.05
Easy	E	2PCC	-	30	30	9.25	8.65	0.89	0.11
Easy	AB	2QFW	-	1	1	1.55	2.79	0.94	0.23
Easy	E	2SIC	-	1	1	1.75	2.32	0.93	0.23
Easy	E	2SNI	-	2	2	3.69	5.97	0.9	0.07
Easy	E	2UUY	-	-	-	-	-	-	-
Easy	A	2VIS	-	10	10	1.73	2.99	0.83	0.14
Easy	E	7CEI	-	10	10	1.71	1.89	0.82	0.19

[a] Subset of 97 structures from the ZDOCK benchmark 3.0. See footnotes of Table S9 for further explanations.

Table S11: Results for unbound global docking with the DrugScore^{PPI}/FRODOCK approach.[a]

Difficulty	Category	PDB ID	Quality_1	Quality_2	Quality_3	i_rmsd	l_rmsd	f_{nat}	f_{not}
Easy	A	1AHW	-	-	504	8.33	9.65	0.23	0.53
Easy	O	1AK4	-	-	-	-	-	-	-
Easy	O	1AKJ	-	-	110	5.31	8.67	0.64	0.69
Easy	E	1AVX	-	6	6	1.69	3.17	0.75	0.12
Easy	E	1AY7	-	562	322	7.69	6.55	0.17	0.84
Easy	O	1B6C	-	43	43	3.6	3.58	0.74	0.48
Medium	A	1BGX	-	-	-	-	-	-	-
Easy	AB	1BJ1	-	195	195	1.79	3.29	0.79	0.06
Difficult	O	1BKD	-	-	12	6.26	5.74	0.17	0.54
Easy	O	1BUH	-	-	1136	5.73	6.4	0.36	0.59
Easy	A	1BVK	-	-	70	7.93	8.62	0.1	0.95
Easy	E	1BVN	-	64	64	2.21	2.36	0.62	0.14
Easy	E	1CGI	-	-	162	9.7	9.11	0.13	0.78
Easy	E	1D6R	-	-	79	6.4	9.62	0.33	0.68
Difficult	O	1DE4	-	-	-	-	-	-	-
Easy	E	1DFJ	-	-	4	5.68	5.52	0.28	0.78
Easy	A	1DQJ	-	-	530	5.68	7.75	0.57	0.37
Easy	A	1E6J	-	438	438	3.46	3.13	0.69	0.6
Easy	E	1EAW	-	1	1	3.47	4.33	0.76	0.29
Difficult	O	1EER	-	-	392	8.24	9.35	0.14	0.7
Easy	E	1EWY	-	524	19	7.33	7.35	0.25	0.85
Easy	E	1EZU	-	-	-	-	-	-	-
Easy	O	1F51	-	-	207	8.38	9.27	0.14	0.82
Easy	O	1FQJ	-	-	892	4.98	6.13	0.52	0.44
Easy	AB	1FSK	-	1	1	1.61	2.03	0.96	0.24
Easy	O	1GCQ	-	-	695	4.72	5.98	0.59	0.54
Easy	O	1GLA	-	-	-	-	-	-	-
Easy	O	1GPW	-	120	120	3.82	4.95	0.85	0.59
Easy	O	1HE1	-	-	21	7.66	7.84	0.18	0.79
Easy	E	1HIA	-	-	89	7.89	9.01	0.26	0.61
Medium	O	1I2M	-	-	255	5.72	6	0.22	0.38
Easy	O	1I4D	-	-	-	-	-	-	-
Easy	AB	1I9R	-	-	-	-	-	-	-
Difficult	O	1IBR	-	-	-	-	-	-	-
Easy	AB	1IQD	-	-	467	5.86	9.9	0.89	0.11
Difficult	O	1IRA	-	-	-	-	-	-	-
Easy	A	1JPS	-	-	-	-	-	-	-
Easy	AB	1K4C	-	-	-	-	-	-	-
Medium	O	1K5D	-	-	263	7.3	9.92	0.17	0.44
Easy	O	1K74	-	311	81	6.44	6.98	0.46	0.59
Easy	O	1KAC	-	1056	383	7.86	9.33	0.19	0.86
Medium	E	1KKL	-	-	-	-	-	-	-
Easy	O	1KTZ	-	-	-	-	-	-	-
Easy	O	1KXP	-	-	358	6.64	6.63	0.28	0.61
Easy	AB	1KXQ	-	-	37	3.44	5.66	0.7	0.29
Medium	E	1M10	-	-	-	-	-	-	-
Easy	E	1MAH	-	13	13	2.7	3.49	0.81	0.39
Easy	O	1ML0	-	39	39	2.47	2.86	0.77	0.34

Easy	A	1MLC	-	23	23	2.17	2.87	0.85	0.49
Medium	O	1N2C	-	-	1724	8.64	9.98	0.2	0.54
Easy	E	1N8O	-	23	23	4	4.9	0.6	0.2
Easy	AB	1NCA	-	1	1	3.03	4.94	0.84	0.25
Easy	AB	1NSN	-	650	650	4.34	4.85	0.63	0.47
Easy	E	1OPH	-	-	-	-	-	-	-
Easy	E	1PPE	-	1	1	2.41	2.55	0.86	0.27
Difficult	E	1PXV	-	-	-	-	-	-	-
Easy	O	1QA9	-	-	-	-	-	-	-
Easy	AB	1QFW	-	292	292	1.72	2.03	0.91	0.18
Easy	E	1R0R	-	87	87	1.43	1.91	0.92	0.21
Difficult	O	1R8S	-	-	-	-	-	-	-
Easy	O	1RLB	-	-	387	3.43	5.17	0.7	0.34
Easy	O	1S1Q	-	415	70	4.68	5.33	0.65	0.6
Easy	O	1SBB	-	-	-	-	-	-	-
Easy	O	1T6B	-	-	901	5.68	8.19	0.48	0.39
Easy	E	1TMQ	-	1746	1746	4.14	4.62	0.51	0.46
Easy	E	1UDI	-	-	75	5.5	5.84	0.36	0.64
Easy	A	1VFB	-	1024	122	8.61	6.84	0.16	0.87
Easy	A	1WEJ	-	649	41	7.33	8.9	0.24	0.8
Medium	O	1WQ1	-	-	154	6.84	9.29	0.28	0.6
Easy	O	1XD3	-	1036	518	8.05	9.69	0.32	0.4
Medium	O	1XQS	-	-	-	-	-	-	-
Difficult	O	1Y64	-	-	-	-	-	-	-
Easy	E	1YVB	-	1	1	2.3	4.8	0.86	0.25
Easy	O	1ZHI	-	-	1095	7.22	9.4	0.35	0.77
Easy	O	2AJF	-	-	-	-	-	-	-
Easy	E	2B42	-	30	30	2.52	3.61	0.73	0.28
Easy	O	2BTF	-	-	630	9.27	9.88	0.14	0.78
Difficult	O	2C0L	-	-	-	-	-	-	-
Medium	O	2CFH	-	178	178	2.43	2.36	0.86	0.42
Easy	A	2FD6	-	-	-	-	-	-	-
Medium	O	2H7V	-	-	-	-	-	-	-
Easy	O	2HLE	-	1077	118	6.21	7.34	0.27	0.57
Difficult	AB	2HMI	-	-	-	-	-	-	-
Easy	O	2HQS	-	-	163	5.12	7.51	0.52	0.52
Medium	O	2HRK	-	-	-	-	-	-	-
Easy	A	2I25	-	-	-	-	-	-	-
Easy	AB	2JEL	-	-	605	8.77	8.99	0.2	0.74
Easy	E	2MTA	-	-	1080	6.59	8.23	0.17	0.86
Medium	O	2NZ8	-	-	-	-	-	-	-
Difficult	O	2OT3	-	-	-	-	-	-	-
Easy	E	2PCC	-	-	1118	7.6	9.86	0.28	0.8
Easy	AB	2QFW	-	-	12	3.51	7.01	0.81	0.29
Easy	E	2SIC	-	215	5	4.56	6.61	0.66	0.45
Easy	E	2SNI	-	-	63	4.88	8.63	0.51	0.32
Easy	E	2UUY	-	-	-	-	-	-	-
Easy	A	2VIS	-	1166	95	5.45	6.22	0.42	0.54
Easy	E	7CEI	-	-	504	8.33	9.65	0.23	0.53

[a] Subset of 97 structures from the ZDOCK benchmark 3.0. See footnotes of Table S9 for further explanations.

Table S12: Results for unbound global docking with the FRODOCK approach.[a]

Difficulty	Category	PDB ID	Quality_1	Quality_2	Quality_3	i_rmsd	l_rmsd	f_{nat}	f_{not}
Easy	A	1AHW	-	288	288	1.41	1.41	0.78	0.07
Easy	O	1AK4	-	-	-	-	-	-	-
Easy	O	1AKJ	-	486	322	8.77	8.11	0.52	0.81
Easy	E	1AVX	-	11	10	3.53	6.24	0.65	0.22
Easy	E	1AY7	-	1	1	3.13	4.45	0.83	0.14
Easy	O	1B6C	-	5	5	3.31	3.33	0.69	0.43
Medium	A	1BGX	-	-	-	-	-	-	-
Easy	AB	1BJ1	-	49	49	1.44	2.51	0.67	0.08
Difficult	O	1BKD	-	-	82	6.07	5.86	0.19	0.51
Easy	O	1BUH	-	1688	476	5.44	5.65	0.37	0.5
Easy	A	1BVK	-	336	13	7.55	8.2	0.24	0.86
Easy	E	1BVN	-	2	2	2.22	2.36	0.62	0.21
Easy	E	1CGI	-	38	19	7.48	7.62	0.21	0.59
Easy	E	1D6R	-	-	96	7.14	8.82	0.14	0.85
Difficult	O	1DE4	-	1597	939	2.99	7.51	0.5	0.43
Easy	E	1DFJ	-	2	1	4.88	5.7	0.66	0.57
Easy	A	1DQJ	-	-	-	-	-	-	-
Easy	A	1E6J	-	17	10	8.94	8.45	0.23	0.82
Easy	E	1EAW	-	41	41	3.26	4.15	0.79	0.24
Difficult	O	1EER	-	-	191	7.18	8.43	0.35	0.58
Easy	E	1EWY	-	1	1	2.65	2.55	0.61	0.48
Easy	E	1EZU	-	197	197	3.37	3.21	0.49	0.13
Easy	O	1F51	-	83	83	4.01	4.01	0.66	0.47
Easy	O	1FQJ	-	-	437	4.52	5.58	0.57	0.35
Easy	AB	1FSK	-	1	1	2.35	2.68	0.85	0.29
Easy	O	1GCQ	-	-	157	6.75	7.98	0.24	0.76
Easy	O	1GLA	-	327	327	4.36	4.72	0.68	0.42
Easy	O	1GPW	-	1	1	2.78	4.04	0.83	0.53
Easy	O	1HE1	-	-	17	8.09	9.23	0.22	0.84
Easy	E	1HIA	-	-	3	8.08	8.79	0.11	0.88
Medium	O	1I2M	-	-	129	4.45	4.83	0.29	0.2
Easy	O	1I4D	-	-	-	-	-	-	-
Easy	AB	1I9R	-	-	-	-	-	-	-
Difficult	O	1IBR	-	-	-	-	-	-	-
Easy	AB	1IQD	-	285	241	6.95	7.42	0.85	0.15
Difficult	O	1IRA	-	-	-	-	-	-	-
Easy	A	1JPS	-	895	562	3.8	7.89	0.57	0.57
Easy	AB	1K4C	-	-	-	-	-	-	-
Medium	O	1K5D	-	-	1307	4.83	6.82	0.26	0.26
Easy	O	1K74	-	214	1	4.95	5.63	0.64	0.52
Easy	O	1KAC	-	529	14	5.6	8.93	0.67	0.64
Medium	E	1KKL	-	-	300	6.74	7.73	0.43	0.55
Easy	O	1KTZ	-	557	557	2.24	2.51	0.74	0.21
Easy	O	1KXP	-	223	167	4.73	7.14	0.4	0.36
Easy	AB	1KXQ	-	4	4	2.92	3.91	0.76	0.23
Medium	E	1M10	-	-	1798	6.28	8.32	0.29	0.32
Easy	E	1MAH	-	1	1	1.8	2.29	0.79	0.26
Easy	O	1ML0	-	10	10	1.99	2.42	0.75	0.28

Easy	A	1MLC	-	46	46	2.17	2.74	0.9	0.48
Medium	O	1N2C	-	-	6	7.59	9.90	0.22	0.51
Easy	E	1N8O	-	101	17	4.43	5.04	0.58	0.26
Easy	AB	1NCA	-	700	700	1.9	2.56	0.68	0.11
Easy	AB	1NSN	-	471	426	5.13	5.84	0.44	0.55
Easy	E	1OPH	-	-	-	-	-	-	-
Easy	E	1PPE	-	1	1	1.67	1.75	0.91	0.24
Difficult	E	1PXV	-	-	42	7.8	6.62	0.1	0.85
Easy	O	1QA9	-	-	-	-	-	-	-
Easy	AB	1QFW	-	376	331	5.87	9.87	0.57	0.48
Easy	E	1R0R	-	29	8	5.68	7.65	0.53	0.53
Difficult	O	1R8S	-	-	-	-	-	-	-
Easy	O	1RLB	-	-	547	4.72	8.26	0.57	0.34
Easy	O	1S1Q	-	303	55	5.89	7.14	0.54	0.65
Easy	O	1SBB	-	-	-	-	-	-	-
Easy	O	1T6B	-	886	646	6.32	8.61	0.45	0.43
Easy	E	1TMQ	-	22	22	3.32	2.82	0.72	0.49
Easy	E	1UDI	-	13	1	6.75	7.08	0.35	0.62
Easy	A	1VFB	-	303	25	6.84	7.93	0.33	0.78
Easy	A	1WEJ	-	36	36	2.01	2.61	0.7	0.14
Medium	O	1WQ1	-	1630	1630	3.95	4.95	0.57	0.38
Easy	O	1XD3	-	40	5	5.64	6.84	0.42	0.34
Medium	O	1XQS	-	-	-	-	-	-	-
Difficult	O	1Y64	-	-	-	-	-	-	-
Easy	E	1YVB	-	1	1	2.11	2.81	0.68	0.31
Easy	O	1ZHI	-	594	153	4.75	8.58	0.61	0.34
Easy	O	2AJF	-	621	621	3.89	3.94	0.55	0.48
Easy	E	2B42	-	1712	2	4.53	6.21	0.55	0.45
Easy	O	2BTF	-	-	457	5.72	6.24	0.29	0.57
Difficult	O	2C0L	-	-	-	-	-	-	-
Medium	O	2CFH	-	6	6	3.55	4.29	0.82	0.43
Easy	A	2FD6	-	-	560	3.93	9.01	0.51	0.47
Medium	O	2H7V	-	-	1615	6.29	8.78	0.6	0.4
Easy	O	2HLE	-	13	13	3.38	3.37	0.53	0.37
Difficult	AB	2HMI	-	-	-	-	-	-	-
Easy	O	2HQS	-	541	59	5.11	5.65	0.47	0.61
Medium	O	2HRK	-	-	308	6.77	7.36	0.6	0.4
Easy	A	2I25	-	-	36	6.52	8.53	0.46	0.31
Easy	AB	2JEL	-	164	55	7.89	8.59	0.22	0.69
Easy	E	2MTA	-	72	20	6.66	7.46	0.38	0.75
Medium	O	2NZ8	-	-	30	9.59	9.62	0.13	0.89
Difficult	O	2OT3	-	-	165	9.12	8.29	0.14	0.62
Easy	E	2PCC	-	734	299	7.4	9.64	0.33	0.75
Easy	AB	2QFW	-	189	113	4.19	9.27	0.69	0.35
Easy	E	2SIC	-	94	19	3.83	5.35	0.65	0.22
Easy	E	2SNI	-	-	-	-	-	-	-
Easy	E	2UUY	-	-	-	-	-	-	-
Easy	A	2VIS	-	465	186	2.77	5.28	0.74	0.2
Easy	E	7CEI	-	288	288	1.41	1.41	0.78	0.07

[a] Subset of 97 structures from the ZDOCK benchmark 3.0. See footnotes of Table S9 for further explanations.

Table S13: Results for unbound knowledge-driven docking with the DrugScore[PPI]/FRODOCK approach.[a]

Difficulty	Category	PDB ID	Quality_1	Quality_2	Quality_3	i_rmsd	l_rmsd	f_{nat}	f_{not}
Easy	A	1AHW	-	-	177	8.45	9.65	0.24	0.53
Easy	O	1AK4	-	-	-	-	-	-	-
Easy	O	1AKJ	-	-	33	5.42	8.67	0.64	0.68
Easy	E	1AVX	-	3	3	1.67	3.17	0.74	0.13
Easy	E	1AY7	-	116	54	7.76	6.55	0.18	0.83
Easy	O	1B6C	-	5	5	3.62	3.58	0.74	0.48
Medium	A	1BGX	-	-	-	-	-	-	-
Easy	AB	1BJ1	-	43	43	1.83	3.29	0.79	0.07
Difficult	O	1BKD	-	-	-	-	-	-	-
Easy	O	1BUH	-	772	217	5.72	6.4	0.36	0.59
Easy	A	1BVK	-	1182	16	8.03	8.62	0.13	0.92
Easy	E	1BVN	-	21	21	2.29	2.36	0.62	0.15
Easy	E	1CGI	-	-	69	9.04	9.11	0.14	0.77
Easy	E	1D6R	-	-	81	5.34	7.53	0.6	0.42
Difficult	O	1DE4	-	-	-	-	-	-	-
Easy	E	1DFJ	-	-	3	5.7	5.52	0.29	0.77
Easy	A	1DQJ	-	-	78	5.7	7.75	0.56	0.38
Easy	A	1E6J	-	21	21	3.56	3.13	0.68	0.6
Easy	E	1EAW	-	1	1	3.76	4.33	0.75	0.3
Difficult	O	1EER	-	1999	356	8.36	9.35	0.15	0.69
Easy	E	1EWY	-	143	12	7.54	7.35	0.26	0.85
Easy	E	1EZU	-	1993	20	5.13	8.75	0.39	0.33
Easy	O	1F51	-	627	85	8.7	9.27	0.15	0.8
Easy	O	1FQJ	-	-	347	5.09	6.13	0.52	0.44
Easy	AB	1FSK	-	1	1	1.69	2.03	0.94	0.25
Easy	O	1GCQ	-	-	575	4.72	5.98	0.59	0.54
Easy	O	1GLA	-	-	1645	8.61	9.25	0.31	0.63
Easy	O	1GPW	-	44	44	3.85	4.95	0.84	0.59
Easy	O	1HE1	-	1303	5	7.67	7.84	0.19	0.78
Easy	E	1HIA	-	-	19	8.05	9.01	0.27	0.6
Medium	O	1I2M	-	-	81	5.93	6	0.23	0.39
Easy	O	1I4D	-	-	-	-	-	-	-
Easy	AB	1I9R	-	-	-	-	-	-	-
Difficult	O	1IBR	-	-	-	-	-	-	-
Easy	AB	1IQD	-	-	62	5.85	9.9	0.82	0.18
Difficult	O	1IRA	-	-	-	-	-	-	-
Easy	A	1JPS	-	1935	1161	3.47	6.54	0.59	0.37
Easy	AB	1K4C	-	-	-	-	-	-	-
Medium	O	1K5D	-	-	83	7.32	9.92	0.17	0.44
Easy	O	1K74	-	76	18	6.42	6.98	0.47	0.59
Easy	O	1KAC	-	273	97	8.16	9.33	0.2	0.85
Medium	E	1KKL	-	446	70	8.22	8.43	0.1	0.91
Easy	O	1KTZ	-	1101	580	6.66	7.46	0.58	0.49
Easy	O	1KXP	-	-	112	6.68	6.63	0.29	0.61
Easy	AB	1KXQ	-	247	4	3.45	5.66	0.7	0.3
Medium	E	1M10	-	-	-	-	-	-	-
Easy	E	1MAH	-	3	3	2.81	3.49	0.8	0.39
Easy	O	1ML0	-	4	4	2.48	2.86	0.76	0.35

Easy	A	1MLC	-	1	1	2.28	2.87	0.84	0.49
Medium	O	1N2C	-	-	174	8.71	9.98	0.21	0.54
Easy	E	1N8O	-	12	12	4.15	4.9	0.59	0.21
Easy	AB	1NCA	-	1	1	3.17	4.94	0.83	0.26
Easy	AB	1NSN	-	77	77	4.55	4.85	0.62	0.47
Easy	E	1OPH	-	-	-	-	-	-	-
Easy	E	1PPE	-	1	1	2.44	2.55	0.85	0.28
Difficult	E	1PXV	-	-	699	7.65	8.87	0.25	0.58
Easy	O	1QA9	-	-	-	-	-	-	-
Easy	AB	1QFW	-	47	47	1.84	2.03	0.9	0.19
Easy	E	1R0R	-	30	30	5.52	1.91	0.91	0.22
Difficult	O	1R8S	-	-	-	-	-	-	-
Easy	O	1RLB	-	790	52	3.54	5.17	0.69	0.35
Easy	O	1S1Q	-	83	12	5.53	5.33	0.65	0.6
Easy	O	1SBB	-	-	-	-	-	-	-
Easy	O	1T6B	-	539	69	6.02	8.19	0.48	0.39
Easy	E	1TMQ	-	216	216	4.17	4.62	0.51	0.47
Easy	E	1UDI	-	1920	9	5.73	5.84	0.36	0.64
Easy	A	1VFB	-	335	45	8.64	6.84	0.18	0.86
Easy	A	1WEJ	-	67	4	7.61	8.9	0.25	0.78
Medium	O	1WQ1	-	-	27	7.17	9.29	0.28	0.59
Easy	O	1XD3	-	151	66	8.25	9.69	0.32	0.4
Medium	O	1XQS	-	-	1261	7.43	8.81	0.22	0.53
Difficult	O	1Y64	-	-	-	-	-	-	-
Easy	E	1YVB	-	1	1	2.31	4.8	0.85	0.25
Easy	O	1ZHI	-	-	288	7.22	9.4	0.36	0.76
Easy	O	2AJF	-	521	373	6.25	6.88	0.26	0.69
Easy	E	2B42	-	12	12	2.63	3.61	0.73	0.28
Easy	O	2BTF	-	-	37	9.37	9.88	0.15	0.76
Difficult	O	2C0L	-	-	843	9.73	9.39	0.3	0.78
Medium	O	2CFH	-	59	59	3.52	2.36	0.85	0.42
Easy	A	2FD6	-	-	714	4.18	8.55	0.45	0.54
Medium	O	2H7V	-	-	726	7.66	9.32	0.33	0.67
Easy	O	2HLE	-	433	59	6.24	7.34	0.28	0.57
Difficult	AB	2HMI	-	-	-	-	-	-	-
Easy	O	2HQS	-	1427	54	5.14	7.51	0.52	0.52
Medium	O	2HRK	-	-	571	10.37	9.91	0.33	0.67
Easy	A	2I25	-	-	1095	9.17	8.07	0.28	0.46
Easy	AB	2JEL	-	-	37	9.35	8.99	0.21	0.73
Easy	E	2MTA	-	-	126	6.68	8.23	0.18	0.85
Medium	O	2NZ8	-	866	678	7.79	8.97	0.26	0.67
Difficult	O	2OT3	-	-	530	6.67	9.19	0.2	0.29
Easy	E	2PCC	-	-	96	10.02	8.42	0.12	0.94
Easy	AB	2QFW	-	1702	8	3.67	7.01	0.8	0.29
Easy	E	2SIC	-	85	4	4.49	6.61	0.66	0.45
Easy	E	2SNI	-	-	35	7.5	8.63	0.51	0.32
Easy	E	2UUY	-	-	-	-	-	-	-
Easy	A	2VIS	-	393	34	5.69	6.22	0.42	0.54
Easy	E	7CEI	-	-	177	8.45	9.65	0.24	0.53

[a] Subset of 97 structures from the ZDOCK benchmark 3.0. Results from one of the three docking runs (see main text for explanation) are shown only. See footnotes of Table S9 for further explanations.

Table S14: Calculation of A_{rel}.

PDB ID[a]	all rmsd [b]	log all rmsd [c]	MW [d]	$A_s^{observed}$ [e]	$A_s^{predicted}$ [f]	A_{rel} [g]
1AHW_l	1.25	0.10	23005	11914	9996	1.19
1AHW_r	0.75	-0.13	46947	19131	17190	1.11
1AK4_l	0.84	-0.07	15126	8585	7268	1.18
1AK4_r	0.37	-0.43	17887	7394	8256	0.90
1AKJ_l	0.62	-0.21	25652	10918	10859	1.01
1AKJ_r	1.13	0.05	43649	18692	16264	1.15
1AVX_l	0.55	-0.26	17865	8308	8248	1.01
1AVX_r	0.52	-0.28	23483	9238	10154	0.91
1AY7_l	0.68	-0.17	10175	5016	5377	0.93
1AY7_r	0.44	-0.35	10571	5619	5536	1.02
1B6C_l	0.34	-0.47	11824	5876	6027	0.97
1B6C_r	0.94	-0.03	37434	15518	14472	1.07
1BGX_l	0.98	-0.01	92091	36273	28685	1.26
1BGX_r	1.62	0.21	46611	18950	17096	1.11
1BJ1_l	0.62	-0.21	22013	11079	9667	1.15
1BJ1_r	-	-	-	-	-	-
1BKD_l	1.34	0.13	18833	8203	8586	0.96
1BKD_r	2.11	0.32	54436	24586	19236	1.28
1BUH_l	1.02	0.01	9190	5528	4977	1.11
1BUH_r	0.99	-0.01	33490	14320	13298	1.08
1BVK_l	0.70	-0.15	14307	6307	6967	0.91
1BVK_r	0.73	-0.13	24762	10110	10571	0.96
1BVN_l	0.55	-0.26	7959	4337	4462	0.97
1BVN_r	0.47	-0.33	55212	17440	19444	0.90
1CGI_l	1.03	0.01	6339	4056	3753	1.08
1CGI_r	0.71	-0.15	25672	10724	10865	0.99
1D6R_l	1.05	0.02	6211	4287	3695	1.16
1D6R_r	0.47	-0.33	23315	9545	10098	0.95
1DE4_l	0.82	-0.09	43032	18569	16089	1.15
1DE4_r	1.21	0.08	143072	46707	40093	1.16
1DFJ_l	0.65	-0.19	13699	6878	6741	1.02
1DFJ_r	1.38	0.14	49009	18385	17760	1.04
1DQJ_l	0.69	-0.16	14307	6306	6967	0.91
1DQJ_r	0.66	-0.18	46107	19039	16955	1.12
1E6J_l	1.48	0.17	8204	5021	4566	1.10
1E6J_r	0.91	-0.04	46576	19161	17086	1.12
1EAW_l	0.60	-0.22	6524	3924	3836	1.02
1EAW_r	0.47	-0.33	26439	10633	11111	0.96
1EER_l	2.38	0.38	45757	22573	16857	1.34
1EER_r	2.78	0.44	18447	10312	8452	1.22
1EWY_l	0.82	-0.08	10685	5085	5581	0.91
1EWY_r	3.32	0.52	62754	26062	21431	1.22
1EZU_l	2.15	0.33	32169	16220	12897	1.26
1EZU_r	0.41	-0.38	23738	9389	10237	0.92
1F51_l	0.80	-0.10	13290	6016	6588	0.91
1F51_r	0.86	-0.06	35319	16402	13846	1.18
1FQJ_l	0.91	-0.04	15679	8722	7470	1.17
1FQJ_r	0.51	-0.30	36206	15058	14110	1.07

1FSK_l	0.67	-0.17	17405	8654	8086	1.07
1FSK_r	-	-	-	-	-	-
1GCQ_l	0.80	-0.10	24495	12638	10484	1.21
1GCQ_r	0.55	-0.26	7740	4187	4368	0.96
1GLA_l	0.50	-0.30	15713	6479	7482	0.87
1GLA_r	0.54	-0.27	55287	18157	19464	0.93
1GPW_l	0.55	-0.26	22339	9022	9775	0.92
1GPW_r	0.89	-0.05	27686	11152	11507	0.97
1HE1_l	0.59	-0.23	19609	9194	8853	1.04
1HE1_r	0.96	-0.02	13889	7152	6812	1.05
1HIA_l	2.34	0.37	5177	3990	3218	1.24
1HIA_r	0.53	-0.27	25586	10370	10837	0.96
1I2M_l	0.42	-0.37	42788	15891	16020	0.99
1I2M_r	1.86	0.27	22833	10397	9939	1.05
1I4D_l	0.68	-0.17	19609	9194	8853	1.04
1I4D_r	0.85	-0.07	45682	21804	16837	1.30
1I9R_l	-	-	-	-	-	-
1I9R_r	0.77	-0.12	47415	16155	17320	0.93
1IBR_l	2.70	0.43	49107	20679	17787	1.16
1IBR_r	1.41	0.15	20925	9732	9301	1.05
1IQD_l	0.35	-0.46	18049	8316	8313	1.00
1IQD_r	-	-	-	-	-	-
1IRA_l	0.53	-0.28	16311	7909	7697	1.03
1IRA_r	6.01	0.78	35612	16766	13933	1.20
1JPS_l	0.87	-0.06	20831	10937	9270	1.18
1JPS_r	0.68	-0.17	46003	18834	16926	1.11
1K4C_l	0.60	-0.22	39854	17153	15178	1.13
1K4C_r	-	-	-	-	-	-
1K5D_l	0.56	-0.25	38223	14480	14703	0.98
1K5D_r	1.44	0.16	38841	16318	14884	1.10
1K74_l	0.77	-0.11	32422	14390	12974	1.11
1K74_r	0.57	-0.24	26329	11490	11076	1.04
1KAC_l	0.71	-0.15	13397	6431	6628	0.97
1KAC_r	0.44	-0.36	19936	8607	8965	0.96
1KKL_l	0.55	-0.26	8938	4579	4873	0.94
1KKL_r	1.08	0.03	48858	22865	17719	1.29
1KTZ_l	0.38	-0.42	11963	6175	6081	1.02
1KTZ_r	1.16	0.07	12724	6975	6373	1.09
1KXP_l	1.85	0.27	50566	23502	18188	1.29
1KXP_r	0.81	-0.09	39636	16562	15114	1.10
1KXQ_l	-	-	-	-	-	-
1KXQ_r	0.43	-0.37	55383	17407	19490	0.89
1M10_l	0.61	-0.21	29549	12817	12091	1.06
1M10_r	0.63	-0.20	23676	9910	10217	0.97
1MAH_l	0.88	-0.06	6764	4106	3943	1.04
1MAH_r	0.62	-0.21	58791	19586	20395	0.96
1ML0_l	1.09	0.04	8151	5508	4543	1.21
1ML0_r	1.52	0.18	81489	31623	26139	1.21
1MLC_l	0.67	-0.18	14307	6306	6967	0.91
1MLC_r	0.84	-0.07	46789	18995	17146	1.11

1N2C_l	4.10	0.61	58716	20887	20375	1.03
1N2C_r	0.45	-0.35	224238	57197	56414	1.01
1N8O_l	0.69	-0.16	15828	8802	7523	1.17
1N8O_r	0.39	-0.41	24830	9863	10593	0.93
1NCA_l	0.29	-0.54	43681	14481	16273	0.89
1NCA_r	-	-	-	-	-	-
1NSN_l	0.94	-0.03	15505	7708	7406	1.04
1NSN_r	-	-	-	-	-	-
1OPH_l	0.30	-0.52	23315	9117	10098	0.90
1OPH_r	0.82	-0.09	41913	17026	15770	1.08
1PPE_l	0.59	-0.23	3257	2630	2262	1.16
1PPE_r	0.40	-0.40	23315	9124	10098	0.90
1PXV_l	0.72	-0.15	13063	7133	6502	1.10
1PXV_r	0.81	-0.09	19991	9086	8984	1.01
1QA9_l	0.80	-0.10	19914	9992	8958	1.12
1QA9_r	0.79	-0.10	20604	10736	9193	1.17
1QFW_l	-	-	-	-	-	-
1QFW_r	1.26	0.10	21252	11053	9412	1.17
1R0R_l	0.62	-0.21	5580	3553	3407	1.04
1R0R_r	0.31	-0.51	27278	9826	11378	0.86
1R8S_l	1.12	0.05	22536	11048	9841	1.12
1R8S_r	0.86	-0.07	20484	9186	9152	1.00
1RLB_l	0.62	-0.21	20055	9681	9006	1.07
1RLB_r	0.74	-0.13	49328	19388	17848	1.09
1S1Q_l	0.74	-0.13	7936	4280	4452	0.96
1S1Q_r	0.66	-0.18	16418	8342	7736	1.08
1SBB_l	0.50	-0.30	28033	11668	11616	1.00
1SBB_r	0.82	-0.08	25850	12380	10922	1.13
1T6B_l	0.50	-0.30	19821	8496	8926	0.95
1T6B_r	1.19	0.08	74897	31161	24515	1.27
1TMQ_l	0.76	-0.12	12686	5994	6359	0.94
1TMQ_r	0.37	-0.44	51095	15955	18332	0.87
1UDI_l	0.88	-0.05	9334	5361	5036	1.06
1UDI_r	0.47	-0.33	25876	10713	10931	0.98
1VFB_l	0.60	-0.22	14307	6627	6967	0.95
1VFB_r	0.43	-0.37	24700	10035	10551	0.95
1WEJ_l	0.44	-0.36	11714	6288	5985	1.05
1WEJ_r	0.75	-0.13	47139	18785	17243	1.09
1WQ1_l	0.78	-0.11	19490	8443	8813	0.96
1WQ1_r	0.85	-0.07	36965	16587	14334	1.16
1XD3_l	0.59	-0.23	7936	4280	4452	0.96
1XD3_r	0.56	-0.25	23585	9734	10187	0.96
1XQS_l	0.65	-0.19	21361	10875	9449	1.15
1XQS_r	2.29	0.36	28343	13037	11714	1.11
1Y64_l	0.98	-0.01	40038	16078	15231	1.06
1Y64_r	10.45	1.02	47317	25630	17293	1.48
1YVB_l	1.33	0.12	12192	6993	6170	1.13
1YVB_r	0.59	-0.23	27152	11055	11338	0.98
1ZHI_l	0.88	-0.06	13302	7494	6592	1.14
1ZHI_r	0.72	-0.14	23502	12050	10160	1.19

2AJF_l	0.95	-0.02	20821	10317	9266	1.11
2AJF_r	0.47	-0.33	69075	25433	23053	1.10
2B42_l	0.54	-0.27	20386	7956	9119	0.87
2B42_r	0.42	-0.37	37016	15229	14349	1.06
2BTF_l	0.56	-0.25	14930	6952	7197	0.97
2BTF_r	1.11	0.05	39636	16563	15114	1.10
2C0L_l	1.23	0.09	13183	6845	6547	1.05
2C0L_r	0.80	-0.10	33183	13554	13205	1.03
2CFH_l	1.43	0.15	16030	8103	7596	1.07
2CFH_r	0.64	-0.19	17536	8613	8133	1.06
2FD6_l	2.73	0.44	29526	14313	12084	1.18
2FD6_r	1.23	0.09	46278	18855	17003	1.11
2H7V_l	0.67	-0.17	19609	9194	8853	1.04
2H7V_r	1.64	0.22	30882	15555	12503	1.24
2HLE_l	0.80	-0.10	15938	8214	7563	1.09
2HLE_r	0.72	-0.15	20449	9809	9140	1.07
2HMI_l	-	-	-	-	-	-
2HMI_r	3.48	0.54	113417	47483	33605	1.41
2HQS_l	0.52	-0.28	12189	5939	6168	0.96
2HQS_r	1.85	0.27	42119	16605	15829	1.05
2HRK_l	0.69	-0.16	11635	6332	5954	1.06
2HRK_r	0.63	-0.20	19560	9784	8837	1.11
2I25_l	0.48	-0.32	14322	6317	6973	0.91
2I25_r	0.49	-0.31	12439	6545	6264	1.04
2JEL_l	0.64	-0.19	9119	4832	4948	0.98
2JEL_r	-	-	-	-	-	-
2MTA_l	0.38	-0.42	11491	5281	5898	0.90
2MTA_r	0.30	-0.52	53042	18653	18861	0.99
2NZ8_l	0.99	0.00	19609	9194	8853	1.04
2NZ8_r	1.69	0.23	35622	16417	13936	1.18
2OT3_l	1.26	0.10	17763	8064	8212	0.98
2OT3_r	0.91	-0.04	28536	14533	11775	1.23
2PCC_l	0.48	-0.32	12063	6395	6120	1.04
2PCC_r	0.34	-0.47	33011	12421	13153	0.94
2SIC_l	0.60	-0.22	11044	5938	5723	1.04
2SIC_r	0.24	-0.61	27538	9891	11460	0.86
2SNI_l	0.49	-0.31	7400	4510	4221	1.07
2SNI_r	0.27	-0.58	27453	9801	11433	0.86
2UUY_l	1.39	0.14	6030	4230	3613	1.17
2UUY_r	0.27	-0.56	23315	9027	10098	0.89
2VIS_l	0.50	-0.30	28952	12724	11905	1.07
2VIS_r	5.08	0.71	46343	19411	17021	1.14
7CEI_l	0.66	-0.18	15032	7741	7234	1.07
7CEI_r	0.62	-0.21	9887	5705	5261	1.08

[a] Subset of 88 structures from the ZDOCK benchmark 3.0. "_r" refers to the receptor protein, "_l" refers to the ligand protein. "-" indicates that no unbound structure was given in the dataset.

[b] All-atom rmsd. Calculated with PyMol [3].

[c] Logarithm of the all-atom rmsd.

[d] Molecular weight in kD. Calculated with Maestro [4].

[e] Observed solvent accessible surface area, for details see ref. [5]. Calculated with Maestro [4].

[f] Predicted solvent accessible surface area calculated as given in eq. 1 from ref. [5].

[g] Relative solvent accessible surface area calculated as given in eq. 2 from ref. [5].

Supplemental Figures

A B

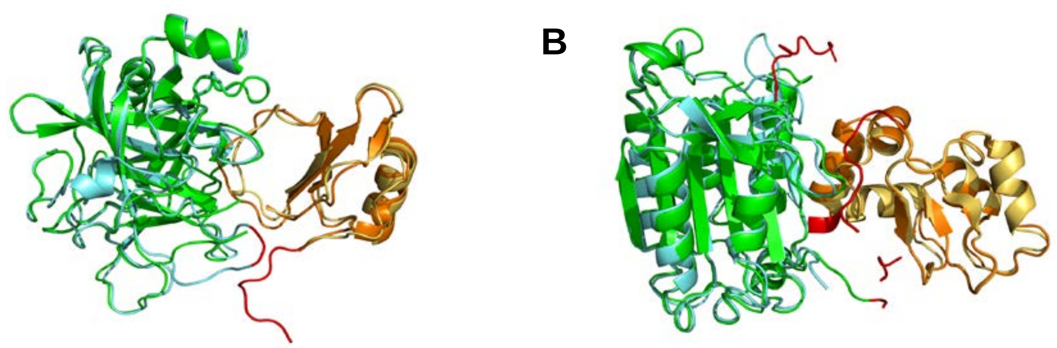

Figure S1: Crystal structures of protein-protein complexes where interfaces are incomplete, i.e., residues are missing: (A) Bovine alpha chymotrypsin in complex with eglin c, (B) Caspase-9 in complex with XIAP-BIR3. Depicted are bound and unbound complexes including the bound receptor (green), unbound receptor (cyan), bound ligand (orange), unbound ligand (yellow), and missing (incomplete) interface parts (red), respectively.

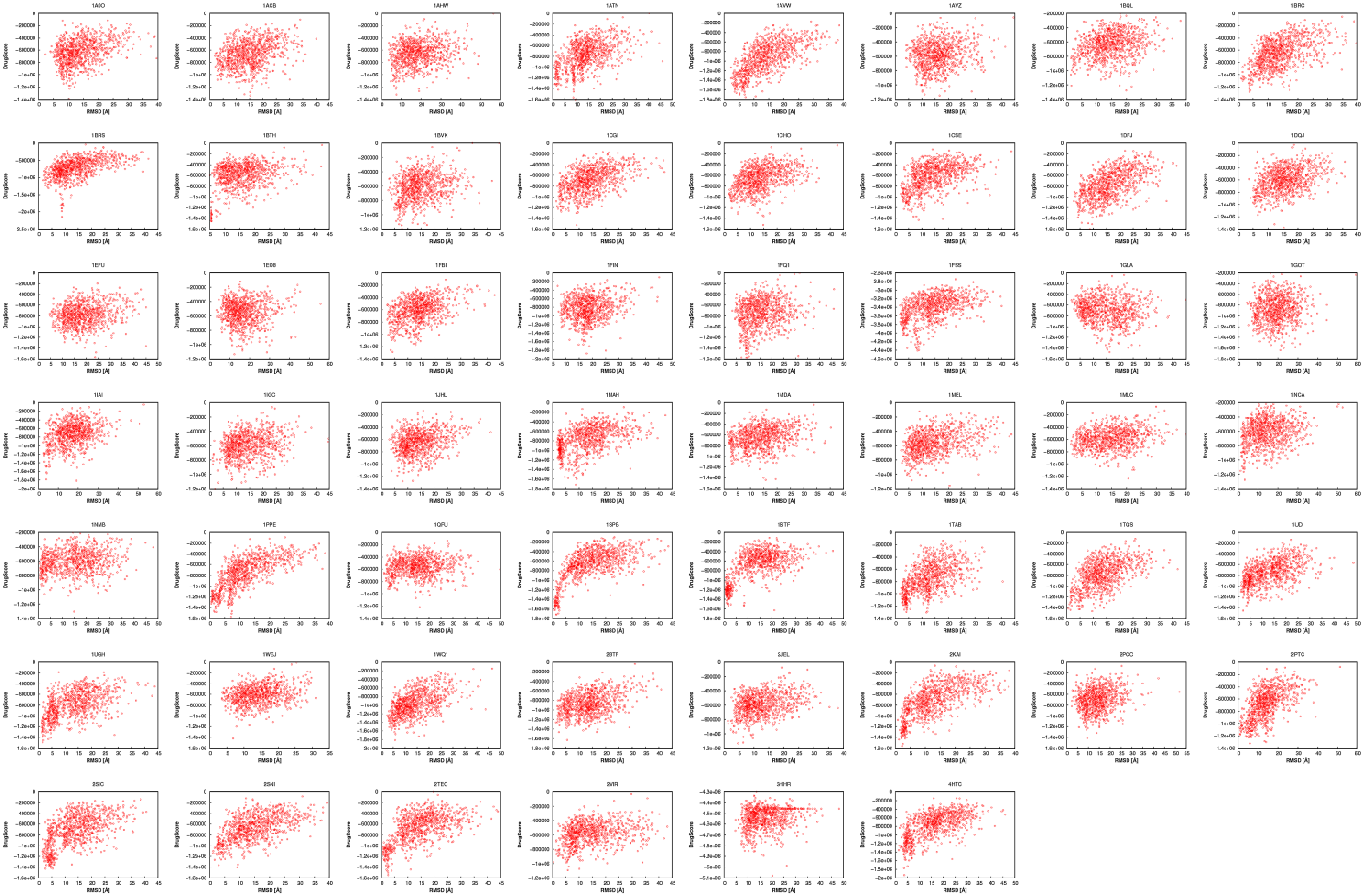

Figure S2: Energy landscapes depicting DrugsScore^{PPI} scores of the decoys of the "unbound perturbation" dataset versus the root mean square deviation of all atoms with respect to the native structure.

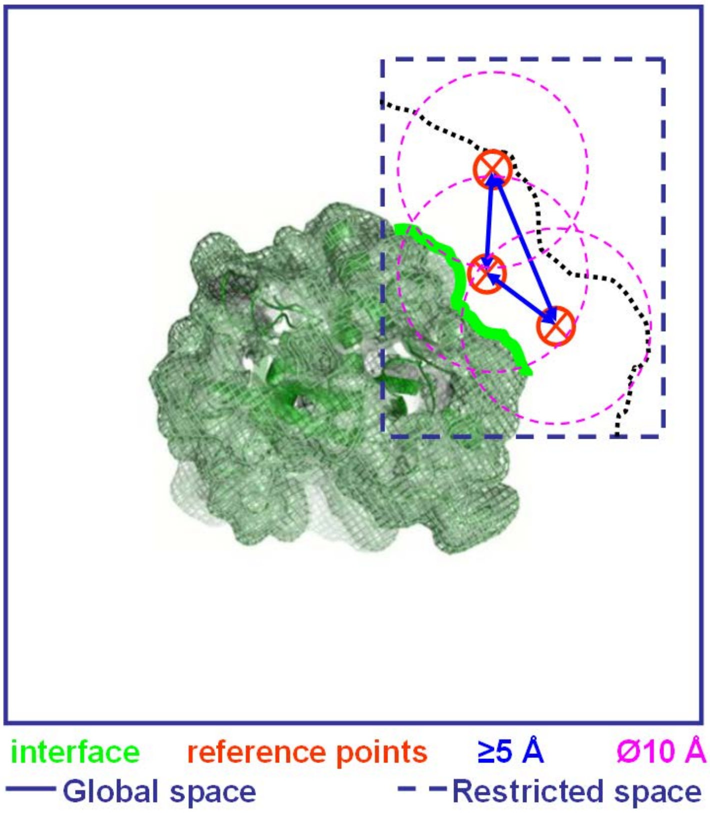

Figure S3: Restriction of the ligand search space for knowledge-driven docking (dark blue dashed line) compared to the global search space (dark blue straight line). For this, a reference point (red) within 5 Å distance (black dotted line) of the receptor interface (green line) is chosen. The search space was then restricted to 10 Å around this reference point (magenta). To minimize the bias by the selected reference point on the docking results, each docking run was repeated three times using a randomly selected reference point where each of the points must be at least 5 Å away (blue arrows) from the other two points.

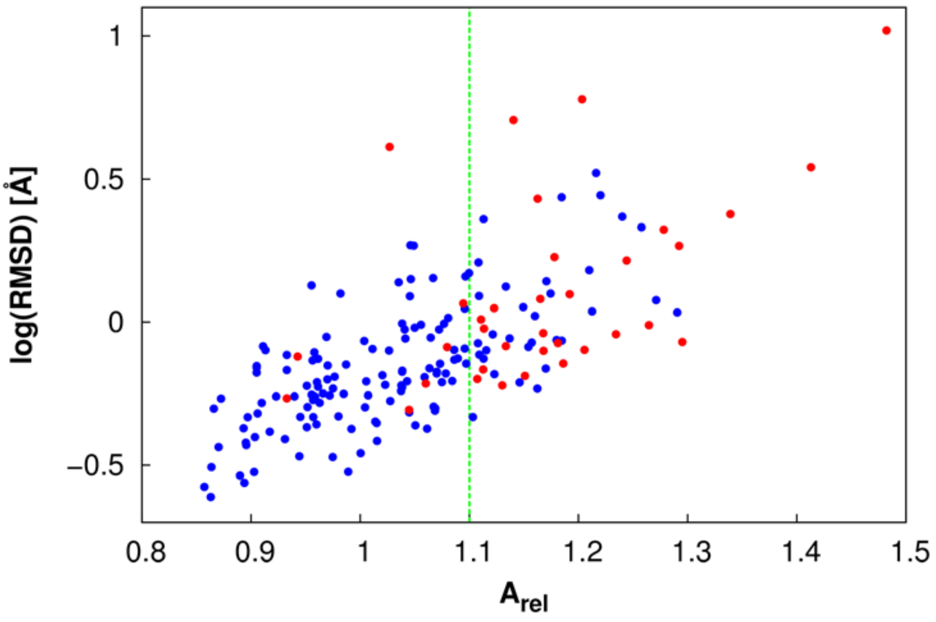

Figure S4: Logarithm of the all-atom rmsd versus calculated A_{rel} (see eq. 2 in ref. [5]) for 88 binding partners in the cleaned version of the ZDOCK benchmark 3.0 where both of the proteins are in the unbound state. The log(rmsd) ~ A_{rel} relation was chosen according to eq. 5 in ref. [5]. Of those unbound knowledge-driven dockings that failed to get a near-native solution with i_rmsd < 10 Å in the top 100, the binding partner with the larger A_{rel} is depicted in red. In 80% of these dockings, at least one of the binding partners had $A_{rel} > 1.1$ (green dashed line).

Predicted	Actual *p*	Actual *n*
p'	**41**	**0**
n'	**7**	**40**

Figure S5: Confusion matrix for two possible outcomes *p* (positive) and *n* (negative) for classifying 88 unbound complexes with respect to whether they can be successfully docked with at least acceptable accuracy in the top 100 predictions using as a criterion that both binding partners have A_{rel} < 1.1. No. of true positives: 41, no. of false positives: 0, no. of false negatives: 7, no. of true negatives: 40.

Supplemental References

1. Hwang H, Pierce B, Mintseris J, Janin J, Weng Z (2008) Protein-protein docking benchmark version 3.0. Proteins 73: 705-709.
2. Gray JJ, Moughon S, Wang C, Schueler-Furman O, Kuhlman B, et al. (2003) Protein-protein docking with simultaneous optimization of rigid-body displacement and side-chain conformations. J Mol Biol 331: 281-299.
3. The PyMOL Molecular Graphics System, version 1.5.0.4, Schrödinger, LLC.
4. Maestro, version 9.1, Schrödinger, LLC.
5. Marsh JA, Teichmann SA (2011) Relative solvent accessible surface area predicts protein conformational changes upon binding. Structure 19: 859-867.
